# Molecular recognition of a Novel thiazolidinedione–morpholine-based ionic liquid salt by HSA: spectroscopic and molecular dynamics analysis

**DOI:** 10.1039/d6ra05468d

**Published:** 2026-08-27

**Authors:** Golshan Golshanian, Mohammad Mehdi Alavianmehr, Mohammad Navid Soltani Rad, Somayeh Behrouz, Mohsen Sorouri, Delara Mohammad-Aghaie

**Affiliations:** a Department of Chemistry, Shiraz University of Technology Shiraz 71555-313 Iran alavianmehr@sutech.ac.ir soltani@sutech.ac.ir

## Abstract

Human serum albumin (HSA) is the most abundant transport protein in human plasma and plays a crucial role in the pharmacokinetic behavior of therapeutic agents. In the present study, the interaction between a newly synthesized thiazolidinedione-morpholine-based ionic liquid salt (TMH-IL) and HSA was systematically investigated using spectroscopic techniques in combination with molecular docking and molecular dynamics (MD) simulations. Steady-state fluorescence spectroscopy revealed concentration-dependent quenching of the intrinsic fluorescence of HSA. Temperature-dependent Stern–Volmer analysis supported a predominantly static quenching mechanism, as evidenced by decreases in both the Stern–Volmer constant (*K*_SV_) and binding constant (*K*_a_) with increasing temperature. The binding constants were on the order of 10^4^–10^5^ M^−1^, indicating moderate affinity between TMH-IL salt and HSA. Thermodynamic analysis yielded negative values of Δ*H* (−73.08 kJ mol^−1^), Δ*S* (−155 J mol^−1^ K^−1^), and Δ*G*, indicating a spontaneous and enthalpy-driven binding process. UV-vis absorption spectroscopy showed concentration-dependent absorption changes, providing complementary evidence for HSA-TMH-IL interaction. FT-IR deconvolution of the amide I region revealed only minor changes in HSA secondary structure, with the α-helical content decreasing from 62.03% to 60.16% upon ligand binding. Consistently, circular dichroism (CD) spectroscopy showed minor reductions in ellipticity at 208 and 222 nm while preserving the characteristic α-helical spectral profile. Three-dimensional fluorescence spectroscopy further indicated perturbation of the Trp214 microenvironment without substantial conformational disruption. Molecular docking predicted Sudlow's site I (subdomain IIA) as the most favorable binding region among the investigated HSA sites. The co-docked anion–cation system yielded the highest ChemPLP score, and interaction analysis revealed hydrophobic contacts and hydrogen-bonding interactions with key residues within the binding cavity. Comparative 150 ns MD simulations of free HSA and the HSA-TMH-IL complex showed that ligand binding did not substantially destabilize the protein or alter its overall compactness. RMSD, RMSF, *R*_g_, SASA, secondary-structure, and hydrogen-bond analyses collectively indicated preservation of the overall HSA architecture, accompanied by limited local conformational rearrangements and persistent protein–ligand interactions. Overall, the combined spectroscopic and computational findings indicate a spontaneous association of moderate affinity between TMH-IL salt and HSA, with molecular docking predicting preferential recognition of Sudlow's site I, while the overall structural integrity of HSA is largely preserved upon ligand binding.

## Abstract 2

Spectroscopic, docking, and molecular dynamics analyses reveal moderate and stable binding of TMH-IL to HSA, preferential recognition at Sudlow’s site I, and preservation of the overall HSA structure.

## Graphical abstract

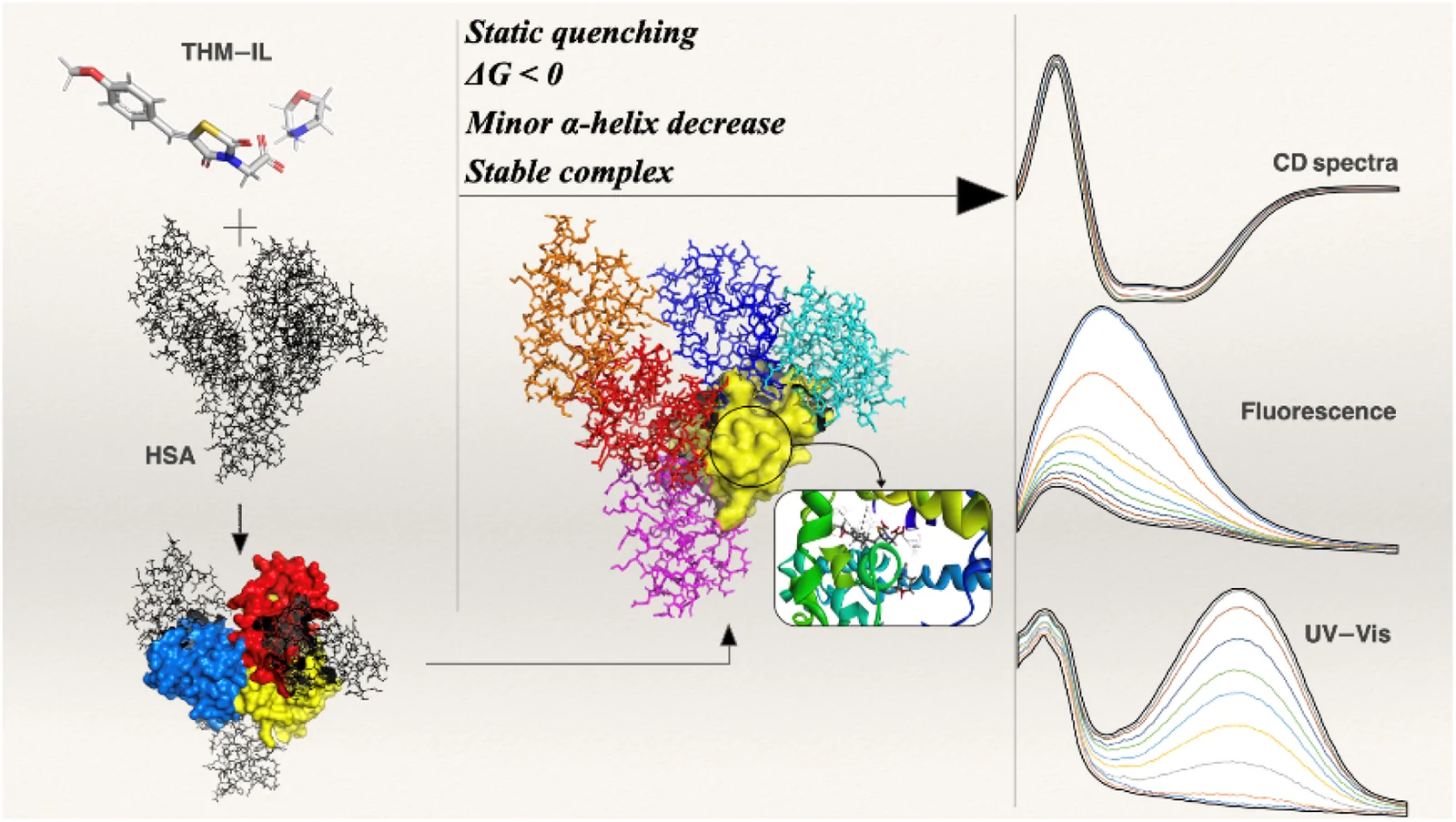

## 1 Introduction

Human serum albumin (HSA) is the predominant protein component of human plasma, accounting for approximately 50–60% of the total circulating protein content and exhibiting a molecular mass of about 66 kDa.^1,2^ Owing to its high plasma abundance, structural flexibility, and multifunctional binding capacity, HSA plays a central role in maintaining physiological homeostasis.^2,3^ In addition to regulating intravascular fluid distribution, HSA functions as the principal transport protein for a wide spectrum of endogenous and exogenous ligands, including fatty acids, hormones, metabolites, and numerous pharmaceutical agents.^3–5^ Moreover, HSA contributes to plasma buffering capacity and displays significant antioxidant properties, primarily through the redox-active Cys34 residue and its ability to scavenge reactive oxygen and nitrogen species.^6–8^ Clinically, serum albumin concentration is widely recognized as a reliable biomarker for evaluating liver function and nutritional status.^9^ These characteristics underscore its indispensable role in survival and justify its therapeutic application in medical conditions such as severe burns, septic shock, sepsis, and hypoalbuminemia.^10^

Beyond its physiological relevance, HSA serves as a highly adaptable carrier protein with the capacity to reversibly bind and transport a diverse array of ligands, including fatty acids, bilirubin, steroid hormones, and numerous endogenous and exogenous therapeutic agents.^11–13^ HSA exhibits remarkable plasma abundance, extended circulatory half-life of approximately 19 days, and the presence of multiple structurally distinct binding cavities collectively render HSA an exceptionally attractive platform for drug delivery and pharmacological modulation.^14–16^ These properties have led to its widespread application in nanomedicine, targeted cancer therapy, and the design of albumin-based strategies aimed at prolonging the systemic half-life of bioactive molecules.^15–18^

**Fig. 1:**
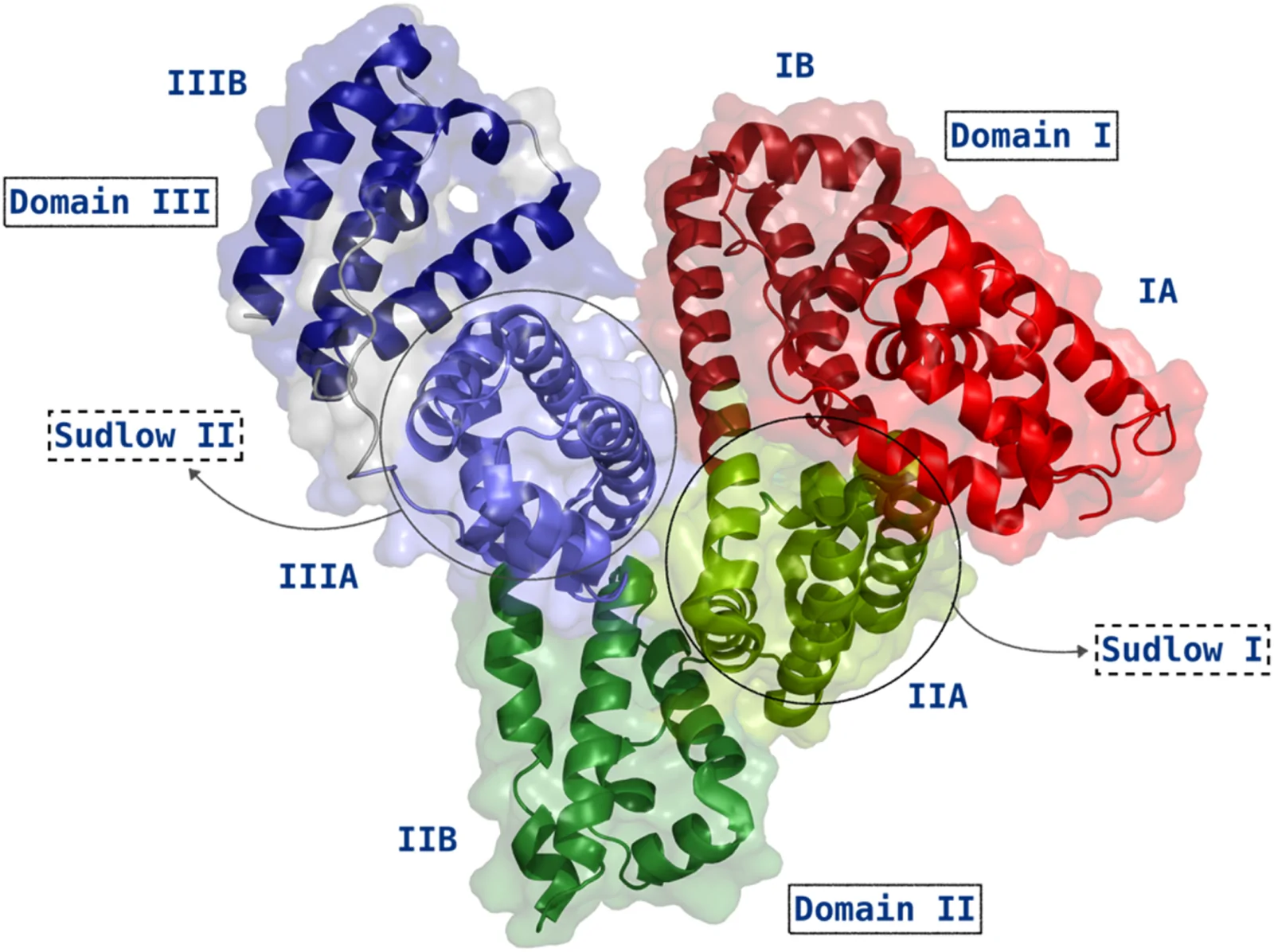
Ribbon representation of HSA generated using PyMOL.

From a structural perspective, HSA consists of three homologous α-helical domains (I, II, and III), each further divided into subdomains A and B, forming a heart-shaped architecture stabilized by multiple disulfide bridges.^19–21^ Among its ligand-binding regions, two major drug-binding pockets-commonly referred to as Sudlow's site I and Sudlow's site II, are primarily located within subdomains IIA and IIIA, respectively.^20,22,23^ These binding sites accommodate a wide spectrum of structurally diverse compounds and play a pivotal role in modulating pharmacokinetic behavior. Drug interactions at these sites significantly affect distribution profiles, bioavailability, elimination rates, plasma half-life, and apparent volume of distribution, thereby influencing therapeutic efficacy and safety.^24–26^ A ribbon representation of the domain organization of HSA is shown in Fig. 1, highlighting the three-domain architecture determined by X-ray crystallography.^17^

Over the past decade, extensive efforts have been devoted to elucidating the molecular basis of HSA–ligand interactions using complementary spectroscopic, chromatographic, and computational methodologies.^27,28^ Spectroscopic approaches, particularly steady-state fluorescence and UV-vis absorption techniques, have proven highly effective in characterizing binding mechanisms, conformational alterations, and thermodynamic signatures of albumin–drug complexes.^29^ For instance, antidepressant and anxiolytic agents such as sertraline and buspirone have been shown to bind spontaneously to HSA, leading to measurable perturbations in secondary and tertiary protein structures.

Similarly, investigations of synthetic cannabinoids have revealed remarkably strong affinities toward Sudlow's site I, often accompanied by competitive displacement and allosteric modulation in the presence of classical site markers including warfarin and ibuprofen.^22^ In parallel, advanced computational frameworks such as molecular docking, molecular dynamics simulations, and site identification by ligand competitive saturation (SILCS) have enabled high-resolution mapping of albumin binding cavities and provided mechanistic insight into ligand recognition and stability within the protein microenvironment. Collectively, these findings highlight not only the structural adaptability of HSA but also its dynamic role in drug binding, transport, and pharmacokinetic modulation.^30–32^

**Fig. 2:**
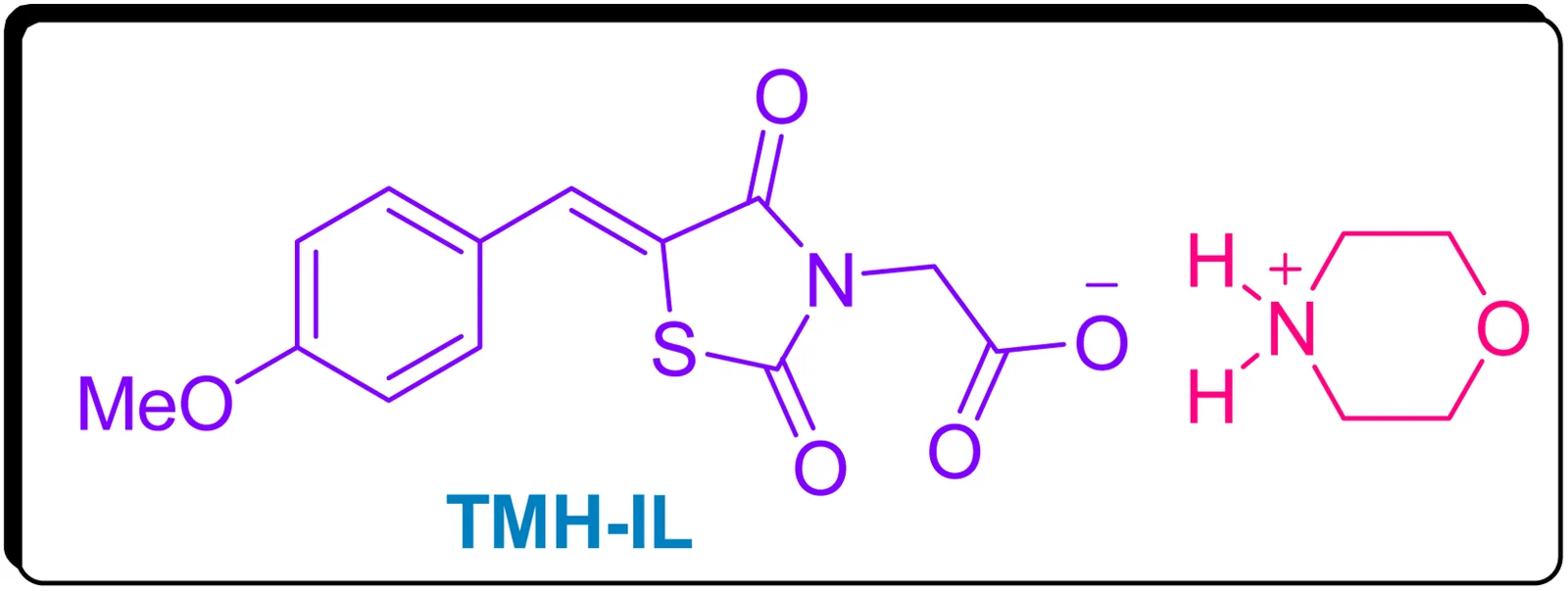
The chemical structure of TMH-IL salt.

The TMH-IL salt investigated in the present work (Fig. 2) belongs to a recently reported series of thiazolidinedione–morpholine hybrid ionic compounds that were designed, synthesized, structurally characterized, and evaluated for antidiabetic activity in streptozotocin-induced diabetic mice.^33^ Several members of this series exhibited significant glucose-lowering activity, supporting their further investigation as potential antidiabetic drug candidates. TMH-IL salt is currently an investigational compound and has not been approved or marketed for clinical use; therefore, its potential application remains at the preclinical research stage. Hence, the present study represents a logical continuation of this previous work, aiming to investigate the interaction of TMH-IL salt with HSA, since HSA binding plays a key role in the transport, distribution, and pharmacokinetic behaviour of drug candidates. Accordingly, elucidating the HSA-binding behaviour of TMH-IL salt is relevant to the broader understanding of drug–protein recognition and provides early molecular-level information on the potential transport and distribution behaviour of this newly developed antidiabetic candidate. The binding behaviour of TMH-IL salt toward HSA was systematically explored using an integrated experimental-computational strategy. Steady-state fluorescence spectroscopy, UV-vis absorption analysis, and infrared (IR) spectroscopy were employed to evaluate binding affinity, conformational perturbations, and microenvironmental changes. These experimental findings were further complemented by molecular docking using GOLD and by molecular dynamics simulations of both unbound HSA and the HSA-TMH-IL complex. This integrated approach provides a comprehensive understanding of the thermodynamic, structural, and dynamic aspects of the HSA-TMH-IL interaction.

It should be noted that although TMH-IL salt is classified as an ionic liquid based on its physicochemical properties, its behaviour in aqueous solution may involve a dynamic dissociation equilibrium between free ions and ion-paired species. Dissolution in water does not necessarily imply complete dissociation, as the extent of ion pairing depends on the specific nature of the constituent ions and their interactions with the surrounding medium. Therefore, in the present study, the term “ionic liquid” refers to the chemical identity and origin of the compound, whereas its interaction with HSA in aqueous solution may involve contributions from both dissociated ionic species and ion-paired forms.

In the present study, all-atom molecular dynamics simulations were performed for both unbound HSA and the HSA-TMH-IL complex to comparatively evaluate the structural stability and dynamic behaviour of the protein in the absence and presence of the ligand. The unbound HSA simulation served as a reference for distinguishing intrinsic protein fluctuations from ligand-induced effects, while the simulation of the HSA-TMH-IL complex enabled assessment of the stability of the docked binding mode and the influence of ligand binding on the conformational dynamics of HSA. Comparative analysis of the trajectories therefore provides a molecular-level framework for interpreting the experimentally observed HSA-TMH-IL interaction and assessing whether ligand binding induces appreciable changes in protein stability and flexibility.

## 2 Materials and methods

HSA (99.8%, CAS NO: 9048-46-8) and other require chemicals were purchased from Merck company. The synthesis of ionic compound, TMH-IL salt (Fig. 2) was carried out due to literature.^33^ The spectroscopic measurements comprising UV-vis and fluorometry were conducted using a Varian Cary 50 Conc and Varian Cary Eclipse fluorescence spectrophotometers, respectively. The IR spectra was carried out using FT-IR spectrometer Brüker, equipped with an ATR accessory. A Metrohm 744 pH meter was used for pH adjustment and monitoring throughout the experiments.

All instruments were calibrated prior to use and operated in accordance with the manufacturer's guidelines. Computational and molecular modelling analyses were performed using Gaussian 16 for quantum chemical calculations, GROMACS 2022 for molecular dynamics simulations, and GOLD 2022.3 for molecular docking studies. Post-processing, visualization, and structural refinement of the obtained complexes were performed using Biovia Discovery Studio 2025 and PyMOL. All computational procedures followed the standard biomolecular simulation protocols to ensure reproducibility and numerical reliability.

### 2.1 Fluorescence spectroscopy

Fluorescence spectroscopy is one of the most sensitive and widely used techniques for investigating protein structure and for characterizing interactions between proteins and small-molecule ligands.^29,34^ In the case of human serum albumin, the intrinsic fluorescence signal arises predominantly from a single tryptophan residue (Trp-214) located within subdomain IIA, corresponding to Sudlow's site I. Due to its location within the primary drug-binding cavity, Trp-214 serves as a sensitive microenvironmental probe that allows detection of ligand-induced conformational changes in the protein structure.^35,36^

Upon excitation at an appropriate wavelength, typically around 280 or 295 nm, the tryptophan chromophore is promoted from the ground electronic state (S_0_) to an excited singlet state (S_1_). The subsequent relaxation process involves vibrational energy dissipation followed by radiative decay, resulting in emission of fluorescence at a longer wavelength than the excitation source a phenomenon known as the Stokes shift. Variations in fluorescence intensity, emission maximum wavelength, or spectral profile provides complementary information regarding possible protein–ligand interactions and ligand-induced structural perturbations.

Fluorescence quenching refers to the attenuation of emission intensity resulting from molecular interactions between an excited fluorophore and a quencher species. This process provides valuable mechanistic information regarding protein–ligand binding, including the nature of the interaction, binding strength, and spatial proximity between the ligand and the intrinsic fluorophore.^37^ In protein systems, quenching behavior is commonly interpreted in terms of two principal mechanisms. Static quenching arises from the formation of a stable, non-fluorescent ground–state complex between the protein and ligand, typically without alteration of fluorescence lifetime. In contrast, dynamic (collisional) quenching occurs when the excited fluorophore undergoes deactivation through diffusional encounters with quencher molecules, leading to a measurable reduction in fluorescence lifetime and enhanced quenching at elevated temperatures due to increased molecular motion.^38^

In human serum albumin, many small-molecule ligands preferentially interact near the Trp-214 residue located within Sudlow's site I, frequently producing quenching patterns consistent with static complex formation. Quantitative interpretation of quenching data is commonly achieved using the Stern–Volmer formalism, which enables determination of Stern–Volmer quenching constants (*K*_SV_), apparent binding constants (*K*_a_), and the number of binding sites (*n*) under appropriate assumptions.^39,40^

In the present investigation, steady-state fluorescence measurements were performed using a Varian fluorescence spectrometer equipped with a 1.0 mL quartz cuvette (path length 1 cm). Spectra were recorded under strictly temperature-controlled conditions at 293.15, 298.15, 303.15, and 310.15 K in order to evaluate the thermodynamic nature of the binding process. Emission spectra were recorded in the wavelength range of 290–500 nm upon excitation at 280 nm, following the excitation at the appropriate wavelength for selective monitoring of the tryptophan residue. Fluorescence titration experiments were carried out by placing a freshly prepared HSA solution (10 µM) into the cuvette and successively adding 15 µL aliquots of the ligand stock solution (0.2 mM). After each incremental addition, the mixture was gently mixed and allowed to equilibrate for approximately 3 min prior to spectral acquisition. A total of nine titration points was recorded, corresponding to a gradual increase in ligand concentration from 0 to 29.27 µM. To minimize possible distortions caused by the inner filter effect (IFE), fluorescence measurements were performed under low-absorbance conditions (*A*_ex_ + *A*_em_ < 0.2). The measured fluorescence intensities were additionally corrected using the standard IFE correction equation (eqn (1)) to ensure accurate quantitative analysis of the quenching process.1
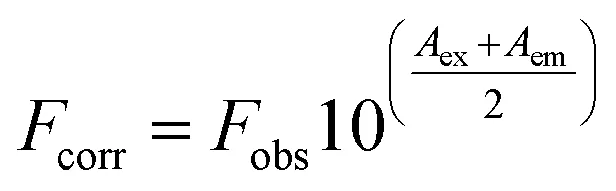
In eqn (1), *F*_corr_ denotes the fluorescence intensity after applying inner filter effect (IFE) correction, whereas *F*_obs_ represents the experimentally measured emission intensity. *A*_ex_ and *A*_em_ correspond to the absorbance values of the sample at the excitation and emission wavelengths, respectively. This correction accounts for attenuation of both the excitation beam and emitted fluorescence within the optical path; thereby, improving the accuracy of quantitative quenching analysis under non-negligible absorbance conditions.^41^

All fluorescence measurements were performed in triplicate under identical experimental conditions, and the results are reported as mean ± standard deviation (SD).

### 2.2 UV-vis absorption spectroscopy

Ultraviolet-visible (UV-vis) absorption spectroscopy was employed as a complementary technique to further elucidate the interaction between the ionic ligand and human serum albumin. This method provides complementary information regarding protein–ligand interactions, alterations in the protein microenvironment, and possible conformational perturbations upon ligand binding.^21,34^

Absorption spectra were recorded using a UV-vis spectrophotometer equipped with a 1.0 cm path length quartz cuvette. A freshly prepared HSA solution (10 µM) was dissolved in the appropriate buffer system, and incremental amounts of the ligand were introduced *via* stepwise titration. The ligand stock solution was added in 15 µL aliquots, leading to a gradual increase in ligand concentration from 0 to 29.27 µM. After each addition, the solution was gently mixed to ensure homogeneity prior to spectral acquisition.

Spectral measurements were collected over the wavelength range of 260–440 nm at ambient temperature. Particular attention was devoted to the characteristic absorption bands of HSA in the vicinity of 210–220 nm (associated with peptide backbone transitions) and around 280 nm (attributed primarily to aromatic amino acid residues, including tryptophan and tyrosine). Changes in absorbance intensity or band position were analyzed to assess potential ground–state complex formation and ligand-induced structural modifications.^34^

### 2.3 FT-IR spectroscopy assessment

Fourier transform infrared (FT-IR) spectroscopy was applied to evaluate potential ligand-induced conformational alterations in human serum albumin upon interaction with TMH-IL salt. FT-IR analysis provides structural information at the level of the peptide backbone and is particularly sensitive to modifications in secondary structure elements.^42,43^

Infrared spectra of native HSA and the HSA-TMH-IL complex were recorded using an FT-IR spectrometer equipped with an attenuated total reflectance (ATR) accessory at room temperature. HSA solutions were prepared in phosphate buffer under conditions identical to those employed in fluorescence and UV-vis measurements to ensure experimental consistency. For complex formation, the protein solution was incubated with TMH-IL salt at a final ligand concentration of 140 µM prior to spectral acquisition, allowing sufficient time for equilibration.

To eliminate spectral interference from the buffer system, the corresponding buffer spectrum was recorded separately under identical conditions and subtracted from all protein-containing spectra before further analysis. Spectra were collected over the range of 4000–500 cm^−1^ at appropriate spectral resolution, and multiple scans were averaged to enhance the signal-to-noise ratio and improve spectral reliability.

Particular attention was devoted to the amide I (≈1600–1700 cm^−1^) and amide II (≈1500–1600 cm^−1^) regions. The amide I band, primarily arising from C=O stretching vibrations of the peptide backbone, is highly sensitive to variations in α-helix, β-sheet, β-turn, and random coil content, whereas the amide II band mainly reflects N–H bending and C–N stretching vibrations.^44,45^ Changes in band position, intensity, or shape were interpreted as indicators of ligand-induced perturbations in protein secondary structure. Where necessary, Gaussian deconvolution of the amide I band was performed to quantitatively estimate secondary structure contributions.

### 2.4 Circular dichroism (CD)

Circular dichroism (CD) spectroscopy is a powerful and sensitive technique for investigating the secondary structure of proteins in solution. In the far-UV region (190–260 nm), the CD signal arises mainly from electronic transitions of the peptide backbone and provides valuable information regarding the α-helical, β-sheet, β-turn, and random coil contents of proteins. Human serum albumin (HSA), which possesses a predominantly α-helical structure, exhibits two characteristic negative bands near 208 and 222 nm.^46^ Variations in the intensity or position of these bands can be used to monitor ligand-induced conformational changes in the protein structure. In the present study, far-UV CD spectroscopy was employed as a complementary technique to fluorescence and FT-IR analyses in order to evaluate the effect of TMH-IL salt binding on the secondary structure of HSA. CD spectra were recorded in the wavelength range of 190–260 nm for HSA (10 µM) in the absence and presence of increasing concentrations of TMH-IL salt under identical experimental conditions. The resulting spectra were analysed to assess possible structural perturbations associated with protein–ligand complex formation.

### 2.5. 3D fluorescence spectroscopy

Three-dimensional fluorescence spectroscopy was employed as a complementary analytical approach to further investigate ligand-induced conformational variations in human serum albumin. Excitation–emission matrix (EEM) spectra provide comprehensive spectral mapping of intrinsic fluorophores and are particularly useful for monitoring microenvironmental changes around aromatic residues.^29,47^

EEM spectra were acquired by systematically scanning both excitation and emission wavelengths within the range of 260–600 nm under temperature-controlled conditions. All instrumental parameters, including slit widths, scanning speed, and photomultiplier voltage, were maintained identical to those applied in steady-state fluorescence quenching experiments to ensure methodological consistency and allow direct comparison between datasets.

In protein systems, a characteristic fluorescence peak was observed at *E*_x_ ≈ 275–280 nm and *E*_m_ ≈ 335–340 nm (Peak I), primarily attributed to the intrinsic fluorescence of aromatic amino acid residues, particularly tryptophan (Trp-214) and tyrosine. Variations in peak intensity, position, and contour distribution were analyzed to assess ligand-induced perturbations in the protein microenvironment and conformational organization. Changes in fluorescence landscape were interpreted as indicators of alterations in the polarity and compactness of the protein microenvironment upon complex formation.^34^

### 2.6 Molecular dynamics simulations

All-atom molecular dynamics (MD) simulations were performed for both unbound HSA and the HSA-TMH-IL complex to comparatively investigate the structural stability and conformational dynamics of HSA under near-physiological conditions. The crystallographic structure of HSA was obtained from the RCSB Protein Data Bank (PDB ID: 7AAI) and used as the initial protein model, while the starting configuration of the HSA-TMH-IL complex was constructed based on the selected docking pose. Prior to the MD simulations, the protein structure was carefully prepared by removing crystallographic artifacts, assigning appropriate protonation states corresponding to physiological pH, and checking for missing atoms or structural inconsistencies. The unbound HSA system was employed as a reference to distinguish intrinsic protein fluctuations from changes associated with TMH-IL salt binding.

The anionic and cationic components of TMH-IL salt were parameterized separately using the LigParGen server within the OPLS-AA force-field framework, with formal charges of −1 and +1, respectively.^48^ The resulting ligand topology and coordinate files were incorporated into the HSA-TMH-IL complex system. Both the unbound HSA and HSA-TMH-IL complex systems were individually placed at the center of cubic simulation boxes and solvated using the explicit TIP3P water model. Appropriate counterions were added to each system to achieve overall charge neutrality. Energy minimization was subsequently performed using the steepest-descent algorithm to remove unfavorable steric contacts and obtain energetically relaxed starting configurations.

Following energy minimization, both systems were equilibrated in two consecutive stages. First, a 100 ps NVT equilibration was performed to stabilize the temperature of the solvated systems, followed by a 1 ns NPT equilibration to achieve appropriate pressure and density relaxation. The temperature was maintained at 310.15 K using the velocity-rescaling (V-rescale) thermostat with a coupling time constant (*τ*_T_) of 0.1 ps, while the pressure was maintained at 1 bar using the Parrinello–Rahman barostat under isotropic coupling conditions. The same equilibration protocol was applied to both unbound HSA and the HSA-TMH-IL complex prior to the production MD simulations.^49–51^

Periodic boundary conditions (PBC) were applied in all three spatial dimensions for both systems to mimic an infinite bulk environment and minimize finite-size effects.^52,53^ All MD simulations for both unbound HSA and the HSA-TMH-IL complex were performed using the GROMACS 2022 simulation package with the OPLS-AA all-atom force field and the explicit TIP3P water model.^54^ Non-bonded interactions were treated using a cutoff distance of 1.0 nm for both van der Waals and short-range electrostatic interactions, while long-range electrostatics were calculated using the Particle Mesh Ewald (PME) method.

Neighbor lists were updated using a cutoff radius of 1.0 nm throughout the simulation. Bond lengths involving hydrogen atoms were constrained using the LINCS algorithm, allowing the use of a 2 fs integration time step. Integration of Newton's equations of motion was performed using the leap-frog algorithm.

Following equilibration, three independent 150 ns production MD simulations, each comprising 75 million integration steps, were performed for both unbound HSA and the HSA-TMH-IL complex under constant temperature and pressure conditions. The resulting trajectories were analyzed using standard GROMACS utilities to comparatively evaluate the structural stability and conformational dynamics of HSA in its free and ligand–bound states. The analyses included backbone root-mean-square deviation (RMSD), root-mean-square fluctuation (RMSF), radius of gyration (*R*_g_), solvent-accessible surface area (SASA), and secondary-structure analysis. Hydrogen-bond analysis was additionally performed for the HSA-TMH-IL complex to assess the persistence of intermolecular hydrogen-bonding interactions during the simulation. Collectively, these analyses were used to evaluate changes in the structural stability, residue-level flexibility, overall compactness, solvent exposure, and secondary-structure behavior of HSA upon TMH-IL salt binding, as well as the dynamic stability of protein–ligand interactions. Unless otherwise stated, comparative MD results are reported as mean values obtained from the three independent trajectories.

**Fig. 3:**
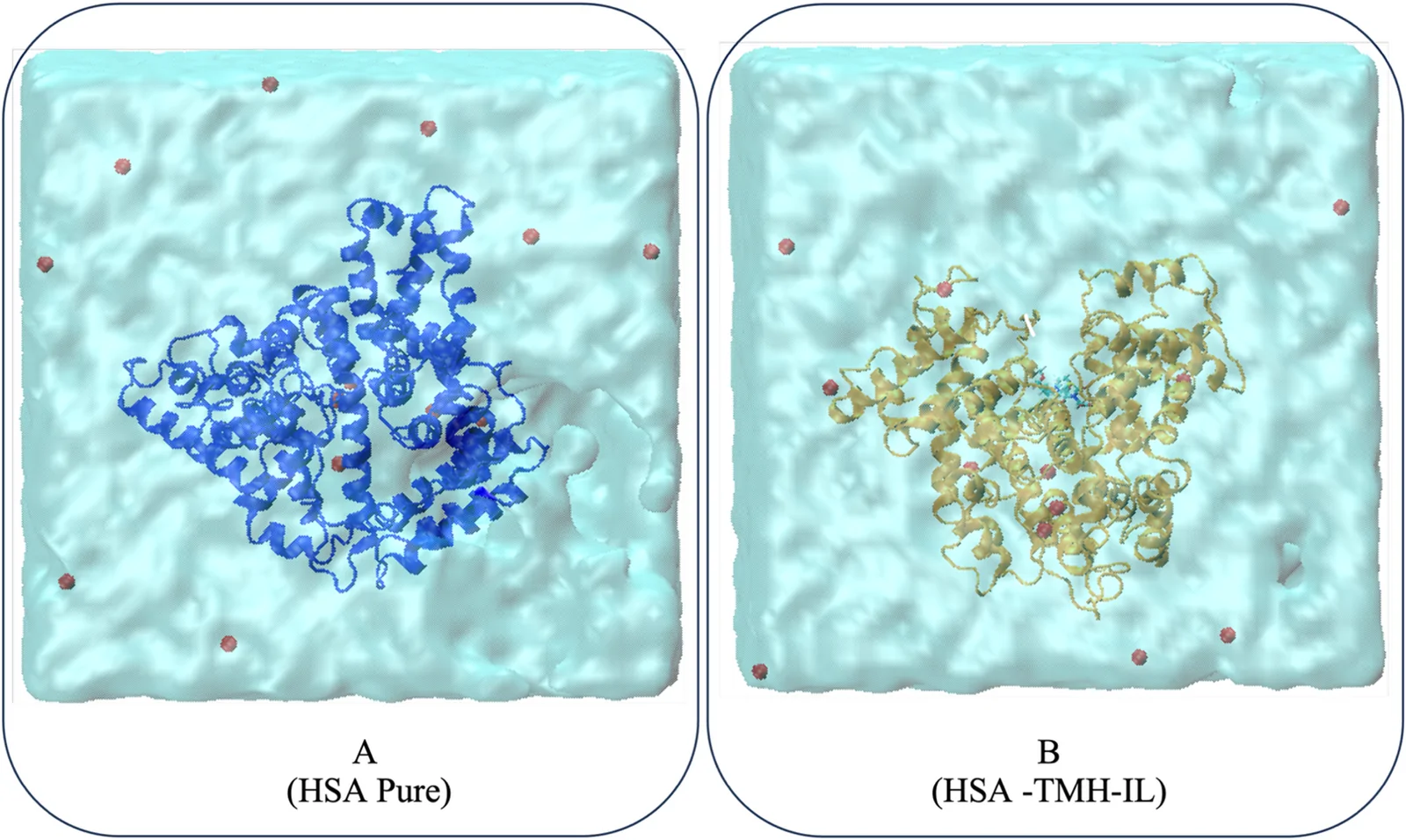
Initial configurations of the molecular dynamics simulation systems: (A) free HSA and (B) the HSA-TMH-IL complex. Both systems were placed in explicit solvent, neutralized with counterions, and subsequently subjected to energy minimization, equilibration, and production MD simulations.

The initial configurations of the free HSA and HSA-TMH-IL simulation systems are shown in Fig. 3.

#### 2.6.1 Root mean square deviation (RMSD)

RMSD values were calculated using eqn (2), which represents the average positional deviation between atomic coordinates of a reference structure and those of a structure at a given simulation time. In this equation, *N* denotes the total number of atoms considered, while *u*_i_ and *v*_i_ correspond to the Cartesian coordinates of atom i in the initial (reference) structure and the instantaneous configuration, respectively.^62^ This parameter provides a quantitative measure of global structural deviation and is widely used to assess the stability of biomolecular systems during molecular dynamics simulations.2
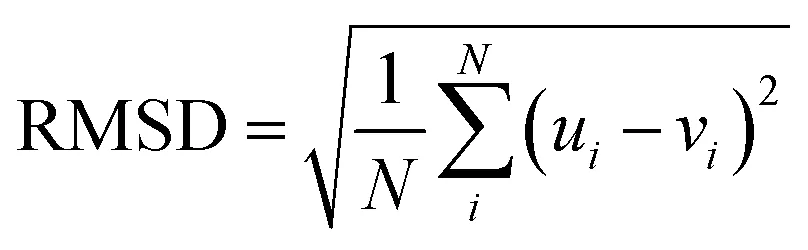


#### 2.6.2 Radius of gyration (*R*_g_)

*R*_g_ values were calculated according to eqn (3) and (4). In these equations, *m*_*i*_ denotes the mass of atom *i*, *r*_*i*_ represents its position vector, *R*_c_ is the centre of mass of the system, and *M* is the total mass of the protein. This formulation provides a mass-weighted measure of the spatial distribution of atoms around the center of mass, and is commonly used to assess protein compactness over time.

3 ∑*m*_*i*_(*r*_*i*_ − *R*_c_) = 0

4
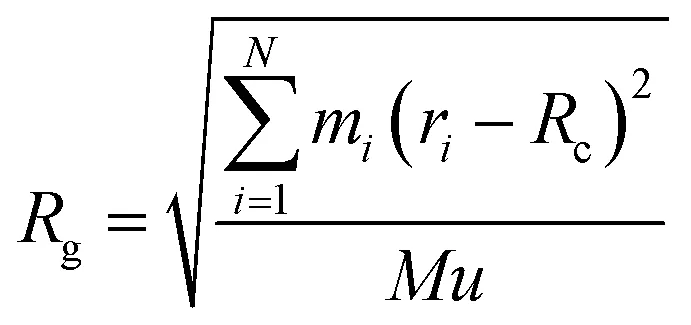


#### 2.6.3 Root mean square fluctuation (RMSF) per residue

The root mean square fluctuation (RMSF) was calculated for both free HSA and the HSA-TMH-IL complex to evaluate residue-level flexibility during the MD simulations. RMSF quantifies the average positional fluctuation of individual residues around their mean positions over the trajectory, thereby providing information on local structural mobility.

### 2.7 Docking study

Molecular docking simulations were conducted to characterize the binding behavior of TMH-IL salt toward HSA and to identify the key intermolecular interactions governing complex formation. The receptor structure employed for docking corresponded to the equilibrated conformation of unbound HSA obtained from molecular dynamics simulation.

To evaluate the contribution of the individual ionic species to the binding process, three docking scenarios were considered, including the isolated cationic fragment, the isolated anionic fragment, and the associated ion pair (cation–anion complex). Each system was docked independently into the protein binding regions to compare their binding affinities and interaction characteristics.

Prior to docking calculations, the ionic ligand structures were fully optimized using density functional theory (DFT) at the B3LYP/6-311++G(d,p) level of theory as implemented in the Gaussian 16 software package.^55–57^ The B3LYP/6-311++G(d,p) level was selected to obtain well-optimized geometries of the ionic species prior to docking. The inclusion of polarization and diffuse functions in this basis set is particularly useful for describing the electronic structure of charged molecular species, while maintaining a reasonable computational cost for the present systems.^58^ The optimized structures were subsequently used as input geometries for molecular docking calculations.

Docking simulations were carried out using the GOLD 2022.3 software package (Cambridge Crystallographic Data Centre, UK), which employs a genetic algorithm to explore ligand conformational flexibility and possible binding orientations within the target protein.

Calculations were performed for the three major ligand-binding regions of HSA, namely Sudlow's site I (subdomain IIA), Sudlow's site II (subdomain IIIA), and site III located in subdomain IB.

The resulting binding poses were ranked according to the ChemPLP scoring function, and the highest-scoring conformations were selected for subsequent interaction analysis. Comparative evaluation of the docking results indicated that the cation–anion ion pair exhibited the most favorable binding characteristics toward HSA, as reflected by its higher ChemPLP score and predicted binding mode relative to the individual ionic fragments.^59,60^

For each docking calculation, 100 independent genetic algorithm (GA) runs were carried out with a search efficiency of 100%. The highest-scoring pose obtained from the docking runs was selected for subsequent interaction and binding mode analyses.^61^

### 2.8 Binding site identification

Potential ligand-binding pockets within the HSA structure were initially identified using an online binding-site prediction server capable of detecting surface-accessible cavities based on geometric and physicochemical characteristics of the protein. This analysis enabled systematic identification of potential binding regions through evaluation of cavity size, depth, and residue composition.

The spatial coordinates of the predicted binding pockets were subsequently extracted and used to define the search regions for docking calculations. Particular attention was given to cavities located within the established ligand-binding regions of HSA, including Sudlow's site I (subdomain IIA) and Sudlow's site II (subdomain IIIA), to ensure the biological relevance of the docking protocol.^29,62^

The integration of computational binding site prediction with molecular docking facilitated more accurate localization of potential ligand-binding regions and reduced the likelihood of identifying non-relevant docking poses. This combined approach enhanced the reliability of binding mode analysis and provided a useful framework for correlating computational predictions with experimental spectroscopic data.

### 2.9 Visualization and interaction analysis

The docked complexes obtained from GOLD simulations were subjected to detailed structural analysis and visualization using Discovery Studio Visualizer 2025 (Dassault Systems BIOVIA).^63^ This software platform was employed to generate two-dimensional (2D) interaction diagrams and to examine the binding orientations of the docked ligands within the HSA binding sites.

Interaction analysis enabled the identification of key amino acid residues involved in ligand recognition and the characterization of the dominant intermolecular forces contributing to complex stabilization. The evaluated interactions included hydrogen bonds, hydrophobic contacts, π–π stacking, π-alkyl interactions, cation-π interactions, and electrostatic interactions. Particular attention was paid to residue-level contacts and the spatial arrangement of the ligand within the binding cavity. The interaction maps generated by Discovery Studio were used to facilitate interpretation of the predicted binding modes and to support comparison between the computational docking results and the experimental spectroscopic observations.

## 3 Results and discussion

### 3.1 Fluorescence spectroscopy assessment

Human serum albumin contains 18 tyrosine, 32 phenylalanine, and a single tryptophan residue (Trp-214), which constitutes the primary intrinsic fluorophore of the protein.^29^ Upon gradual addition of TMH-IL salt, a systematic decrease in the emission intensity of HSA was observed, suggesting effective fluorescence quenching of the Trp-214 microenvironment.

The emission spectrum of free TMH-IL salt recorded under identical experimental conditions exhibited negligible fluorescence within the investigated spectral window (Fig. S1, SI), confirming that the observed changes in fluorescence intensity originated predominantly from HSA rather than ligand emission interference. To quantitatively evaluate the quenching process, the Stern–Volmer equation was employed:5
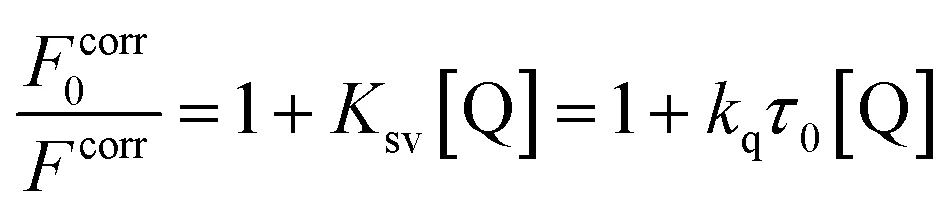
where *F*_corr_ and *F*^corr^_0_ denote the fluorescence intensities of HSA in the absence and presence of quencher, respectively, and [Q] represents the ligand concentration. The Stern–Volmer constant (*K*_sv_) describes the overall quenching efficiency, while the bimolecular quenching rate constant (*k*_q_) is obtained by correlating *K*_sv_ with the intrinsic excited-state lifetime of the fluorophore (*t*_0_). The fluorescence lifetime of serum albumin in the absence of quencher is typically reported in the range of 8–10 ns.^64,65^

Fluorescence measurements were carried out at four temperatures (293.15, 298.15, 303.15, and 310.15 K) using nine ligand concentrations at each temperature to elucidate the quenching mechanism. According to Stern–Volmer theory, the temperature dependence of the quenching constant provides insight into the nature of the interaction. A decrease in *K*_sv_ with increasing temperature generally indicates static quenching due to reduced stability of the ground–state complex at elevated temperatures, whereas an increasing *K*_sv_ trend suggests diffusion-controlled dynamic quenching. In the present study, the observed decrease in *K*_sv_ with increasing temperature indicates that the fluorescence quenching of HSA by TMH-IL salt predominantly follows a static quenching mechanism.

**Fig. 4:**
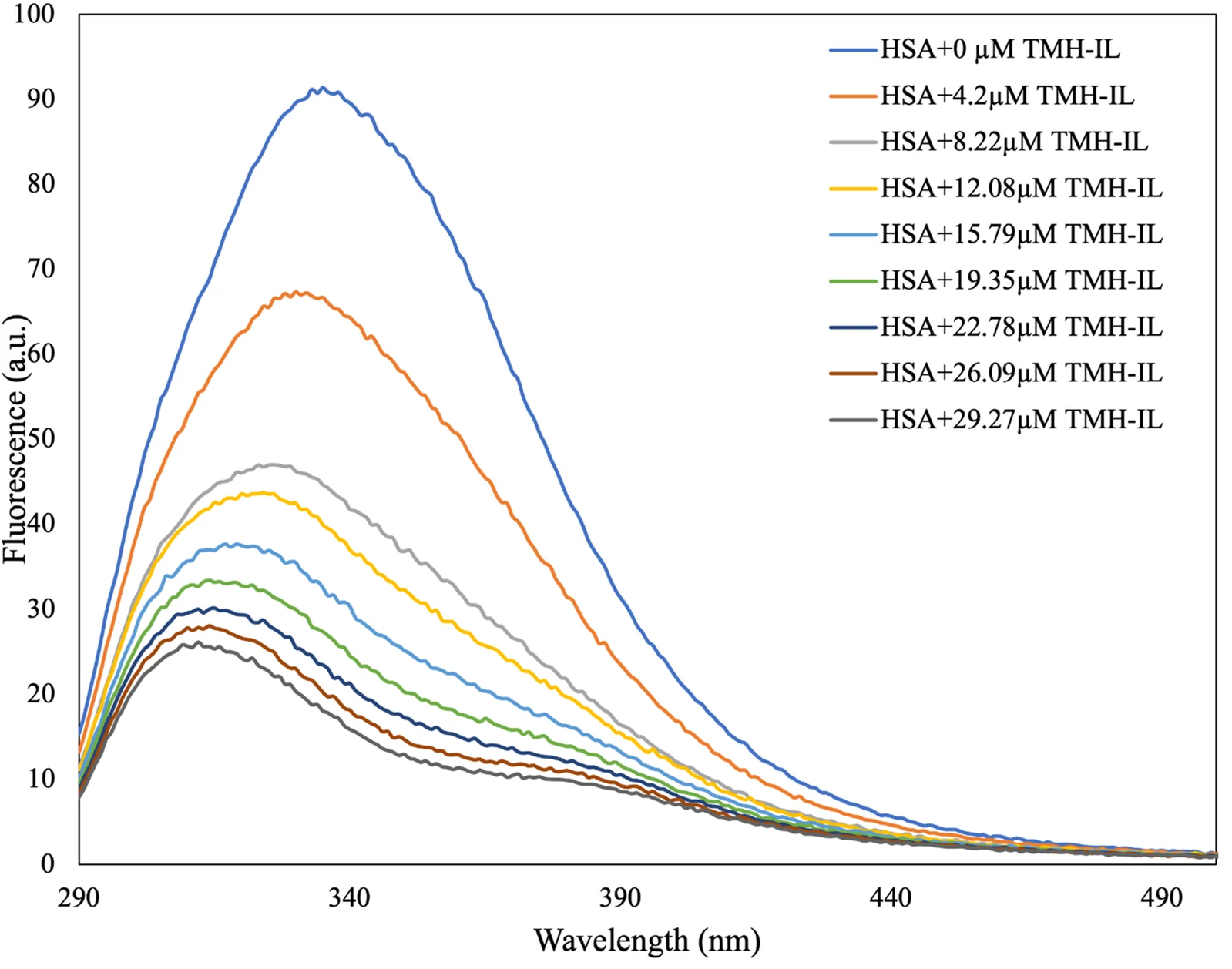
Fluorescence emission spectra of HSA (10 µM) in the presence of increasing concentrations of TMH-IL salt (0–29.27 µM) at 310.15 K.

Steady-state fluorescence emission spectra of HSA were recorded upon excitation at 280 nm in the absence and presence of increasing concentrations of TMH-IL salt. As shown in Fig. 4, gradual addition of the ligand led to a significant decrease in fluorescence intensity. At the highest ligand concentration, an apparent blue shift of the emission maximum from approximately 337 nm to 313 nm was observed. However, UV-vis absorption measurements revealed that TMH-IL salt exhibits noticeable absorption in the 330–360 nm region, overlapping with the emission band of HSA. Therefore, the observed spectral displacement may partially arise from re-absorption effects and preferential attenuation of the long-wavelength emission tail rather than solely from a genuine change in the microenvironment surrounding Trp-214.

It should be emphasized that excitation at 280 nm excites both tyrosine and tryptophan residues; however, Trp-214 is the predominant contributor to the intrinsic fluorescence band centered near 337 nm. A true hypsochromic shift of 25 nm in Trp emission would imply substantial alteration of its microenvironment. However, UV-vis measurements revealed that TMH-IL salt exhibits noticeable absorbance in the 330–360 nm region, partially overlapping with the emission tail of HSA. Therefore, the observed spectral displacement is more plausibly attributed to differential attenuation of longer-wavelength emission components due to re-absorption effects, rather than to a genuine change in the intrinsic emission energy of Trp-214.

The absence of new emission bands, together with the progressive decrease in fluorescence intensity, indicates an interaction between HSA and TMH-IL salt. The predominantly static contribution to the quenching process is supported primarily by the temperature dependence of the Stern–Volmer and binding constants and by the magnitude of the calculated bimolecular quenching rate constants.

To minimize ambiguity associated with excitation of multiple intrinsic fluorophores and potential spectral distortion, quantitative quenching analyses were performed by monitoring fluorescence intensity at a fixed wavelength corresponding to the native Trp emission maximum.

**Fig. 5:**
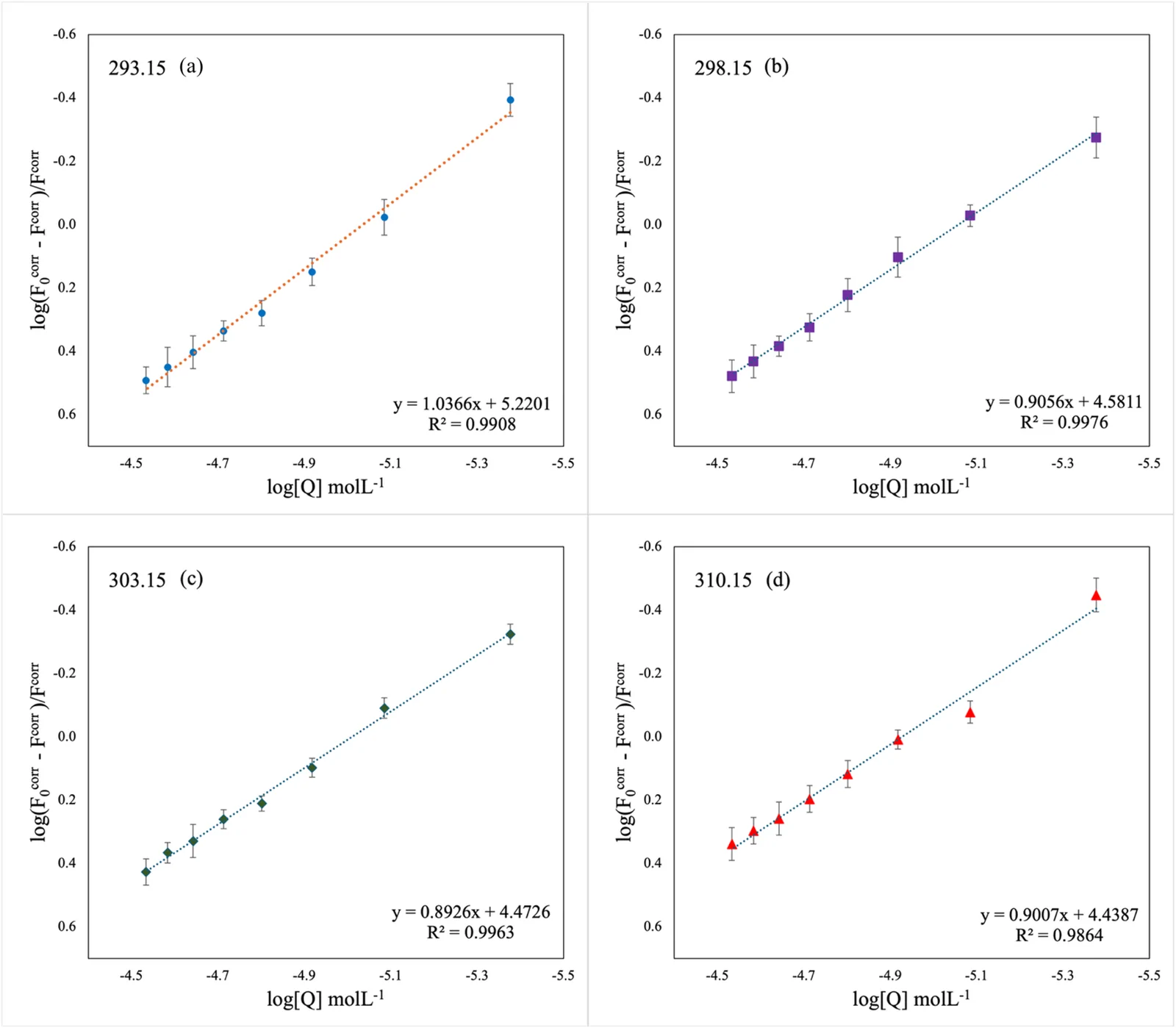
Stern–Volmer plots for the fluorescence quenching of (HSA, 10 µM) by TMH-IL salt (0–29.27 µM) at four different temperatures: (a) 293.15 K, (b) 298.15 K, (c) 303.15 K, and (d) 310.15 K. Data are presented as mean ± SD from three independent measurements (*n* = 3).

Fig. 5 illustrates the Stern–Volmer plots obtained at four different temperatures. A systematic reduction in the Stern–Volmer constant (*K*_sv_) was observed with increasing temperature. Such temperature dependence is consistent with a predominantly static quenching mechanism, because increasing temperature can weaken the non-fluorescent association responsible for the observed quenching.

The analysis was performed using fluorescence data corrected for inner filter effects under low-absorbance conditions to ensure reliability of the calculated parameters. In agreement with the observed trend in *K*_sv_, the binding constant (*K*_a_) also exhibited a decreasing pattern with increasing temperature. This behaviour indicates reduced HSA-TMH-IL binding affinity at higher temperatures and provides further support for a predominantly static contribution to the quenching process.

The consistent temperature dependence of the quenching and binding constants supports the internal coherence of the fluorescence analysis and indicates that the observed fluorescence attenuation contains a predominantly static contribution.

Table 1 lists the Stern–Volmer quenching constants (*K*_sv_) obtained at different temperatures. The corresponding bimolecular quenching rate constants (*k*_q_) were calculated using the average fluorescence lifetime of HSA. Notably, the calculated *k*_q_ values were on the order of 10^13^ M^−1^ S^−1^, significantly exceeding the maximum diffusion-controlled quenching rate in aqueous media (≈2 × 10^10^ M^−1^ S^−1^).^29^

**Table 1:** Stern–Volmer quenching constants (*K*_sv_) for the interaction of TMH-IL salt with HSA at different temperatures. Values are expressed as mean ± standard deviation (*n* = 3)

*T* (°K)	*K*_sv_ (1 × 10^5^ M^−1^)	*k*_q_ (1 × 10^13^ M^−1^ S^−1^)	*R* ^2^
293.15	1.101 ± 0.03	1.101 ± 0.03	0.994
298.15	1.053 ± 0.03	1.053 ± 0.03	0.998
303.15	0.935 ± 0.03	0.935 ± 0.03	0.993
310.15	0.786 ± 0.03	0.786 ± 0.03	0.990

Since diffusion-controlled dynamic quenching cannot reasonably account for *k*_q_ values of this magnitude, the results strongly support a predominantly static contribution to the observed fluorescence quenching.

Under near-physiological conditions, the binding equilibrium between the ligand and HSA was further analyzed using eqn (6).6
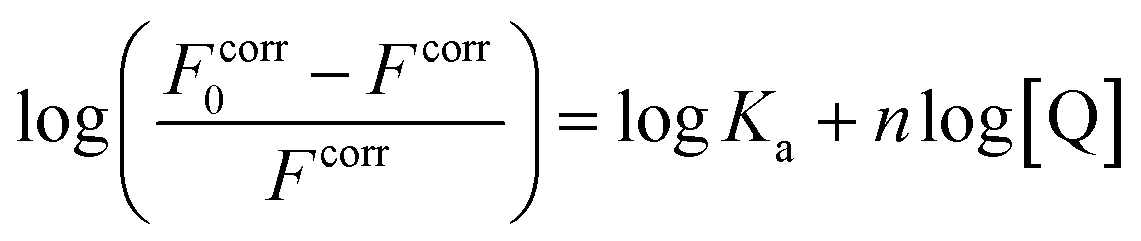


To further characterize the interaction between TMH-IL salt and HSA, the binding equilibrium was analysed using the double-logarithmic form of the modified Stern–Volmer equation. In this representation, the binding constant (*K*_a_) reflects the affinity between ligand and protein, while the parameter *n* corresponds to the apparent number of binding sites per HSA molecule.

**Fig. 6:**
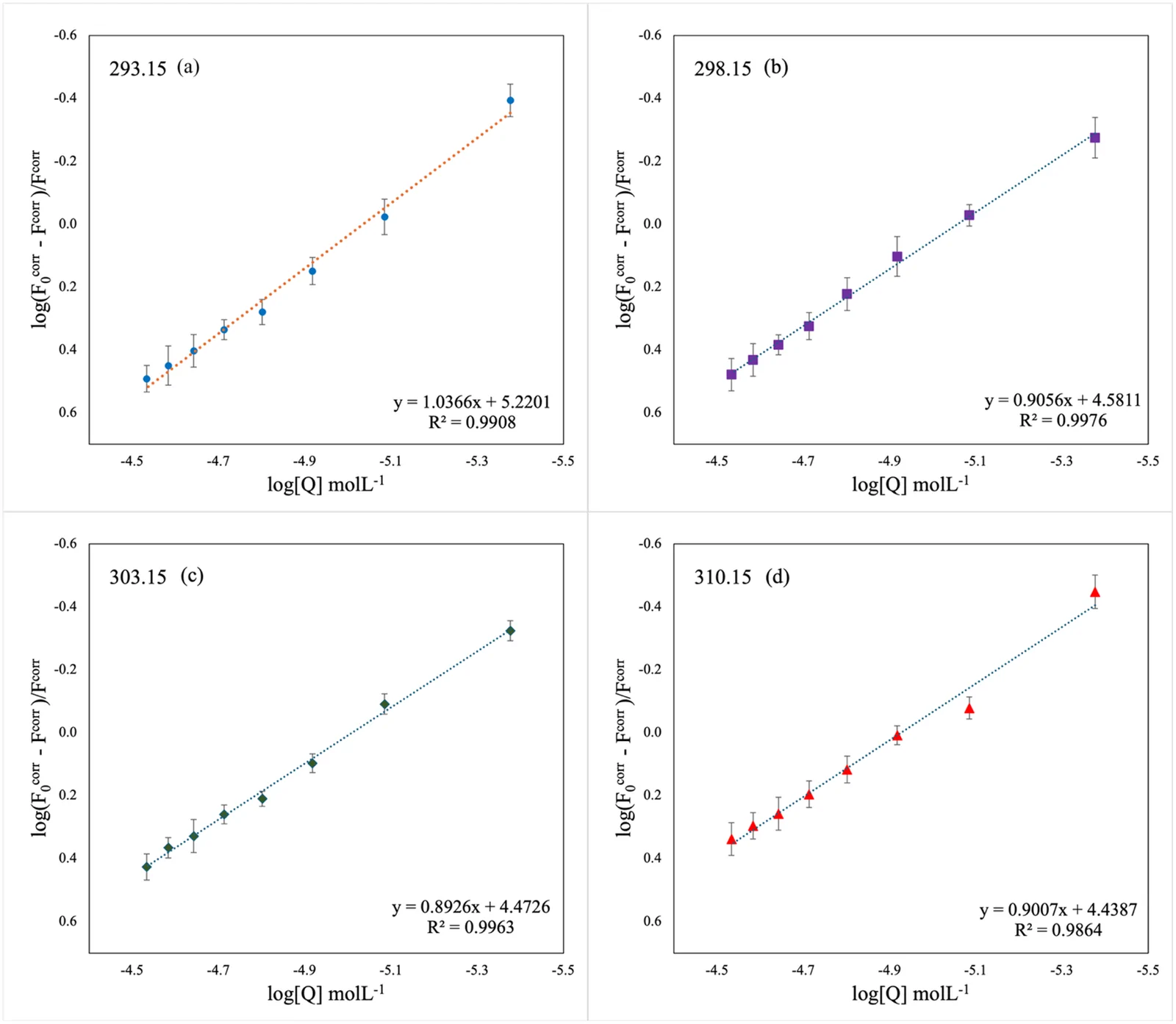
Double logarithmic plots of log[(*F*^corr^_0_ − *F*^corr^)/*F*^corr^] *versus* log[*Q*] for the fluorescence quenching of HSA(10 µM) by TMH-IL salt (0–29.27 µM) at four different temperatures: (a) 293.15 K, (b) 298.15 K, (c) 303.15 K, and (d) 310.15 K. Data are presented as mean ± SD from three independent measurements (*n* = 3).

The resulting modified Stern–Volmer plots are shown in Fig. 6, demonstrating satisfactory linearity across the studied concentration range.

Table 2 summarizes the calculated binding constants (*K*_a_) and the corresponding binding site parameters (*n*) at different temperatures. The magnitude of *K*_a_ provides quantitative insight into the affinity between TMH-IL salt and HSA, where higher values reflect stronger protein–ligand association and consequently a greater fraction of bound drug under equilibrium conditions.^39^

**Table 2:** Binding parameters (*K*_a_, *n*) of TMH-IL salt with HSA at different temperatures. Values are expressed as mean ± standard deviation (*n* = 3)

*T* (°K)	*K*_a_ (1 × 10^4^ M^−1^)	*n*	*R* ^2^
293.15	16.60 ± 0.04	1.04	0.991
298.15	3.81 ± 0.04	0.91	0.997
303.15	2.97 ± 0.04	0.89	0.996
310.15	2.74 ± 0.04	0.90	0.986

In the present system, the calculated *K*_a_ values were on the order of 10^4^–10^5^ M^−1^, indicating a moderate yet meaningful interaction between TMH-IL salt and HSA. The observed decline in *K*_a_ with increasing temperature indicates weakening of the HSA-TMH-IL association at elevated temperatures and is consistent with a predominantly static quenching mechanism. The binding site parameter (*n*) close to unity suggests the presence of a single dominant binding site for TMH-IL salt on HSA.

To gain deeper insight into the driving forces governing the interaction, thermodynamic parameters were determined from the temperature dependence of *K*_a_ using the Van't Hoff equations (eqn (7) and (8)).7
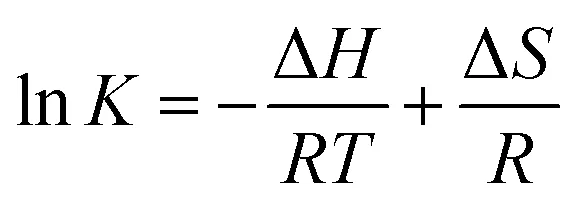

8 Δ*G* = −*RT*ln *K* = Δ*H* − *T*Δ*S*

In the Van't Hoff framework, *R* represents the universal gas constant (8.3145 J mol^−1^ K^−1^). A linear relationship was obtained by plotting ln *K*_a_ against 1/*T*, where the slope and intercept correspond to −Δ*H*/*R* and Δ*S*/*R*, respectively. The calculated thermodynamic parameters are summarized in Table 3.

**Table 3:** Thermodynamic binding parameters for the interaction of TMH-IL salt with HSA at different temperatures. Δ*H* and Δ*S* values were obtained from Van't Hoff analysis and are temperature-independent

*T* (K)	Δ*H* (kJ mol^−1^)	Δ*S* (J mol^−1^ K^−1^)	Δ*G* (kJ mol^−1^)
293.15			−27.65
298.15	−73.08	−155	−26.87
303.15			−26.10
310.15			−25.01

The negative enthalpy change (Δ*H* = −73.08 kJ mol^−1^) indicates that the interaction between TMH-IL salt and HSA is exothermic in nature. Such a favorable enthalpic contribution suggests that specific noncovalent interactions significantly contribute to complex stabilization. The simultaneously negative entropy change (Δ*S* = −155 J mol^−1^ K^−1^) implies a reduction in configurational freedom upon binding, consistent with the formation of a more ordered protein–ligand assembly.

The relatively large magnitude of Δ*H* points to the cumulative effect of multiple interaction forces rather than a single dominant contribution. These likely include hydrogen bonding, electrostatic interactions involving the ionic components, and van der Waals contacts within the binding pocket. The molecular docking results support this interpretation, revealing extensive polar contacts alongside hydrophobic interactions at the proposed binding site.

Similar thermodynamic analyses have been reported for pioglitazone–HSA interactions, where hydrogen bonding and van der Waals interactions were identified as the dominant stabilizing forces.^66^ The present findings therefore indicate that TMH residue shares similar non-covalent interaction patterns while exhibiting a distinct binding affinity.

Furthermore, the negative Gibbs free energy (Δ*G*) values obtained at all investigated temperatures confirm the spontaneous nature of the binding process. Although the association remains thermodynamically favorable across the studied temperature range, the gradual decline in *K*_a_ with increasing temperature indicates reduced binding affinity at elevated temperatures.

Comparison with previously reported thiazolidinedione-based systems further highlights the distinct HSA-binding behavior of TMH-IL salt. The parent 2,4-thiazolidinedione was reported to bind HSA with an association constant of 1.69 × 10^3^ M^−1^ at 298 K, with an approximately 1 : 1 binding stoichiometry and a predominantly static quenching mechanism.^67^ In comparison, TMH-IL salt exhibited a considerably higher association constant (3.81 × 10^4^ M^−1^ at 298.15 K), while retaining an approximately 1 : 1 binding stoichiometry. These findings indicate that structural modification of the thiazolidinedione scaffold and incorporation of the morpholinium ionic component substantially enhance its interaction with HSA. Rosiglitazone has likewise been reported to interact with serum albumin through a predominantly static quenching mechanism; however, that study employed BSA rather than HSA and reported more pronounced alterations in albumin secondary structure.^68^ In contrast, the FT-IR and CD results obtained for TMH-IL salt indicate only limited perturbation of HSA secondary structure. Related HSA interactions have also been investigated for 2,4-thiazolidinedione–phenothiazine molecular hybrids.^69^ In addition, recently reported 4-thiazolidinone derivatives were primarily investigated as inhibitors of HSA glycation rather than through conventional equilibrium binding analysis; nevertheless, their docking results demonstrated structure-dependent HSA recognition.^70^ Collectively, these comparisons demonstrate that TMH-IL salt exhibits a distinct combination of enhanced albumin recognition and relatively limited structural perturbation compared with previously investigated thiazolidinedione-based systems.

### 3.2 UV-vis absorption spectroscopy

UV-vis spectroscopy was employed to complement the fluorescence results and examine spectral changes associated with the interaction of HSA with TMH-IL salt. Serum albumin exhibits characteristic absorption features in the ultraviolet region that arise mainly from electronic transitions of the peptide backbone and aromatic residues such as Trp, Tyr, and Phe. In particular, absorption near 280 nm is predominantly associated with aromatic residues, while transitions of the peptide backbone occur at shorter wavelengths.^71^ Therefore, alterations in absorbance intensity or wavelength position can reflect modifications in the local environment of these chromophoric groups.

UV-vis titration experiments were performed using a 10 µM HSA solution with increasing concentrations of TMH-IL salt (0–29.27 µM) at ambient temperature. The absorption spectra were recorded in the range of 260–440 nm. The native HSA spectrum exhibited a characteristic absorption band near 280 nm, mainly attributed to π–π* transitions of aromatic amino acid residues such as Trp, Tyr, and Phe.

**Fig. 7:**
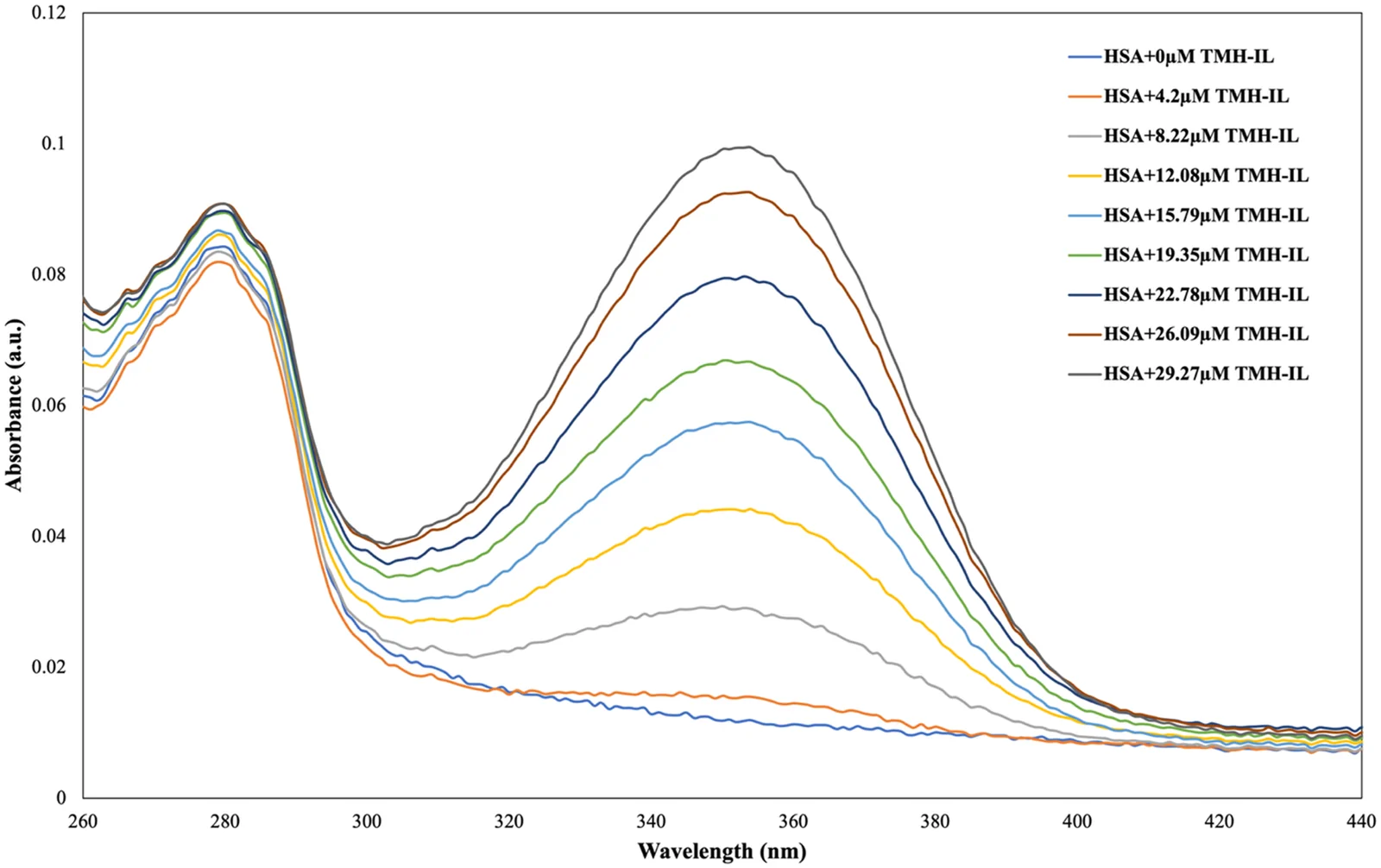
UV-vis absorption spectra of HSA (10 µM) with increasing concentrations of TMH-IL salt (0–29.27 µM) at 298.15 K.

As illustrated in Fig. 7, gradual addition of TMH-IL salt resulted in concentration-dependent changes in the absorption spectrum. An increase in apparent absorbance was observed around 280 nm without a significant shift in the band position. Because TMH-IL salt itself contributes to the absorption in the investigated spectral region, the observed increase cannot be attributed exclusively to structural changes in HSA.

A progressive increase in absorbance was also observed in the 330–360 nm region, which is mainly attributable to the intrinsic absorption of TMH-IL salt. Accordingly, the overlap between the absorption bands of TMH-IL salt and HSA should be considered when interpreting these spectra and does not, by itself, demonstrate formation of a discrete protein–ligand complex.

Overall, the absorption changes indicate that the spectral properties of the system are modified upon mixing HSA with TMH-IL salt. However, because no distinct isosbestic point was observed, the UV-vis data alone cannot unequivocally establish formation of a discrete ground–state complex. These findings are therefore considered complementary to the fluorescence results rather than independent proof of the static quenching mechanism.

### 3.3 FT-IR spectroscopy

Fourier transform infrared (FT-IR) spectroscopy was utilized to assess structural perturbations in HSA following interaction with TMH-IL salt. After appropriate baseline correction and buffer subtraction, the spectra of native HSA and the corresponding HSA-TMH-IL complex were comparatively analysed.

**Fig. 8:**
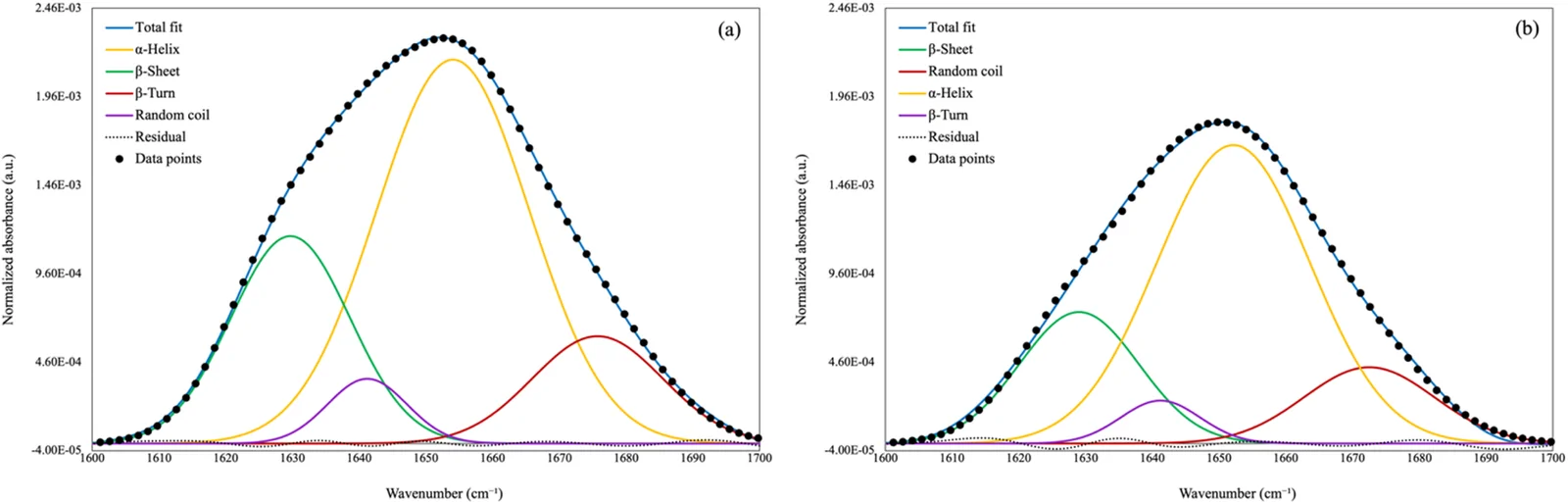
Gaussian deconvolution of the amide I region (1600–1700 cm^−1^) of (a) free HSA and (b) HSA in the presence of TMH-IL salt after buffer subtraction. Experimental data points (●), total fitted curve (solid line), individual Gaussian components assigned.

As illustrated in Fig. 8a (free HSA) and Fig. 8b (HSA-TMH-IL), noticeable and reproducible changes were primarily observed within the amide I region (1600–1700 cm^−1^), which is highly sensitive to alterations in protein secondary structure. In contrast, the amide II band (∼1540 cm^−1^) exhibited only minor variations in intensity without significant frequency shifts, suggesting that the overall backbone framework remained largely preserved upon ligand binding.

Given the high sensitivity of the amide I band to protein secondary structure, quantitative analysis was restricted to this spectral region using Gaussian curve fitting. The amide I envelope was deconvoluted into four principal components attributed to α-helix (1645–1659 cm^−1^), β-sheet (1615–1635 and 1680–1695 cm^−1^), random coil (1636–1644 cm^−1^), and β-turn (1661–1679 cm^−1^) structures. Representative experimental spectra, fitted envelopes, individual Gaussian contributions, and residual distributions are presented in Fig. 8a and b. The residuals were randomly distributed around zero, indicating satisfactory fitting quality and reliability of the deconvolution procedure.

The calculated secondary structure contents are summarized in Table 4. Native HSA exhibited α-helix, β-sheet, random coil, and β-turn contributions of 62.03%, 22.42%, 4.29%, and 14.72%, respectively. Following interaction with TMH-IL salt, these values shifted to 60.16%, 20.96%, 4.65%, and 13.31%.

**Table 4:** Percentages were obtained from the normalized area under each Gaussian component relative to the total amide I band area

Secondary structure	Wavenumber range (cm^−1^)	HSA 50 µM (%)	HSA 50 µM + 140 µM TMH-IL (%)
α-Helix	1645–1659	62.03	60.16
β-Sheet	1615–1635	22.42	20.96
Random coil	1636–1644	4.29	4.65
β-Turn	1661–1679	14.72	13.31
Total	1600–1700	100	100

The observed reduction in α-helical content from 62.03% to 60.16% (a decrease of 1.87 percentage points) together with a slight increase in random coil content (from 4.29% to 4.65%), indicates localized structural rearrangements within the protein backbone upon ligand binding. In addition, the β-sheet content decreased from 22.42% to 20.96%, while the β-turn contribution showed a minor reduction from 14.72% to 13.31%. These variations suggest subtle perturbations in the secondary structural organization of HSA, possibly arising from ligand-induced modifications in intramolecular hydrogen-bonding interactions. Nevertheless, the overall secondary structure profile remained largely preserved, indicating that TMH-IL salt binding induces only moderate conformational adjustments rather than extensive unfolding or denaturation of the protein.

Consistent with this interpretation, the amide II region displayed only minor intensity variations without significant frequency shifts, supporting preservation of the global protein framework.

Taken together, the FT-IR results indicate that interaction with TMH-IL salt induces only limited changes in the secondary structure of HSA while largely preserving the structural integrity of the protein. These observations complement the fluorescence and thermodynamic results, which indicated static quenching and spontaneous, enthalpy-driven interaction, respectively.

### 3.4 Circular dichroism (CD) spectra analysis

**Fig. 9:**
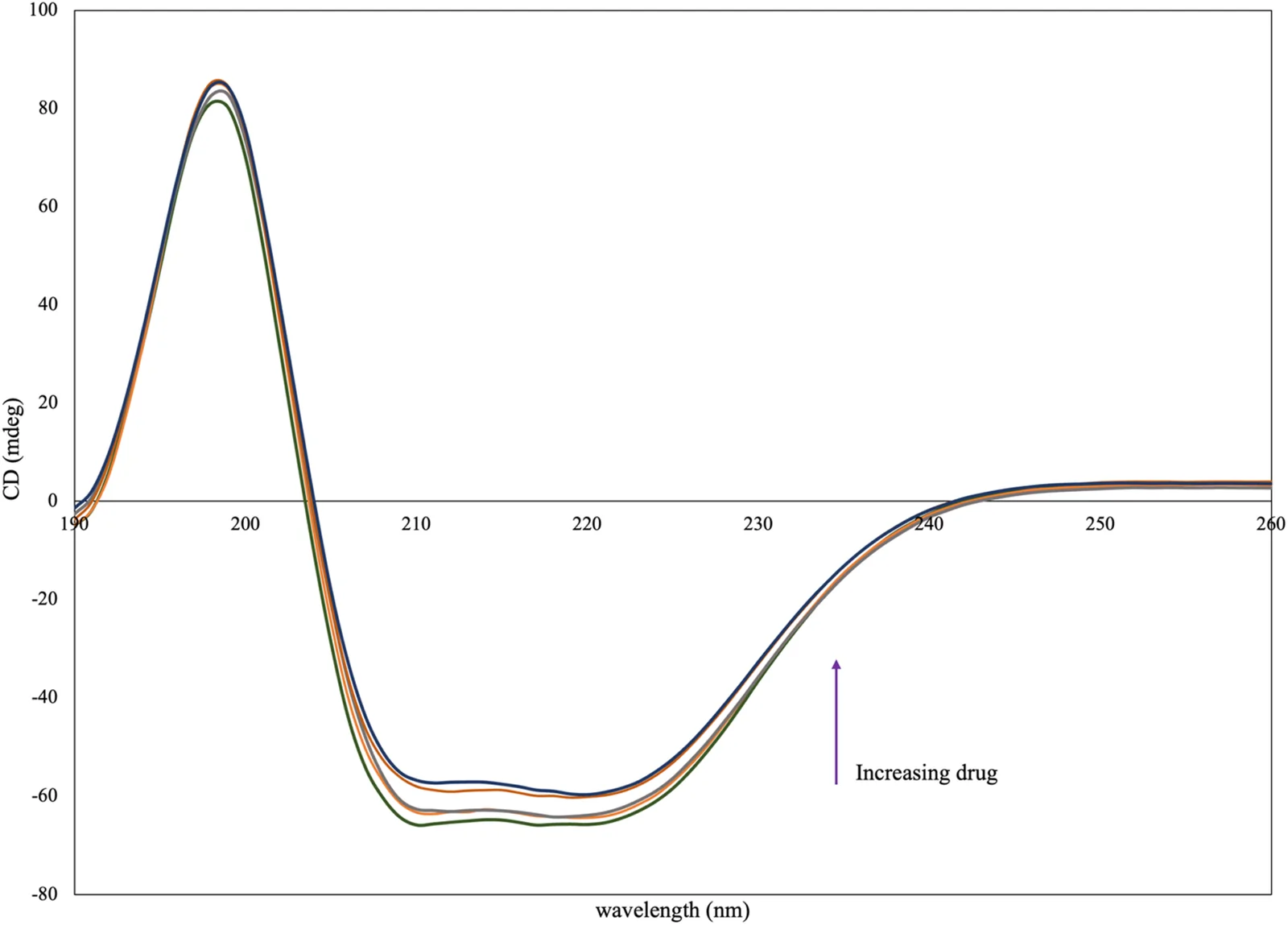
Far-UV CD spectra of HSA (10 µM) recorded in the presence of increasing concentrations of TMH-IL salt (0–29.27 µM) at 298.15 K.

The far-UV CD spectra of HSA in the absence and presence of increasing concentrations of TMH-IL salt are presented in Fig. 9. The spectrum of native HSA displayed two characteristic negative bands centred at approximately 208 and 222 nm, confirming the predominance of α-helical secondary structure in the protein. As shown in Table 5, gradual addition of TMH-IL salt resulted in a slight decrease in the intensity of both negative bands without causing any significant shift in their spectral positions. The ellipticity at 208 nm decreased from −60.20 to −57.73 mdeg, corresponding to a reduction of approximately 4.10%, whereas the ellipticity at 222 nm decreased from −64.48 to −62.40 mdeg, corresponding to a reduction of approximately 3.2%. The observed decrease in ellipticity indicates that the interaction of TMH-IL salt with HSA induces minor perturbations in the secondary structure of the protein. However, the overall spectral profile remained essentially unchanged, demonstrating that the native α-helical framework of HSA was largely preserved after ligand binding. The absence of appreciable wavelength shifts and the persistence of the characteristic α-helical bands further suggest that TMH-IL salt does not induce extensive unfolding or denaturation of the protein. Instead, the interaction appears to promote only limited local conformational rearrangements around the binding region.

**Table 5:** Ellipticity values at 208 and 222 nm for HSA in the absence and presence of TMH-IL salt

Sample	Ellipticity at 208 nm (mdeg)	Ellipticity at 222 nm (mdeg)
HSA	−60.20	−64.48
HSA + TMH-IL	−57.73	−62.40

Furthermore, the CD results are in good agreement with the FT-IR deconvolution analysis of the amide I region. FT-IR measurements revealed a slight decrease in α-helical content from 62.03% in free HSA to 60.16% in the HSA-TMH-IL complex. Therefore, both spectroscopic techniques consistently demonstrate that TMH-IL salt binding induces only subtle modifications in the secondary structure of HSA while preserving the overall structural integrity of the protein. These findings provide additional evidence for protein–ligand complex formation and support the conclusion that TMH-IL salt interacts with HSA without causing significant global structural changes.

Only slight alterations in the secondary structure of HSA were observed, in agreement with previous reports for rosiglitazone and 2,4-thiazolidinedione, suggesting that binding of thiazolidinedione derivatives generally does not induce extensive conformational changes in serum albumin.

### 3.5. 3D fluorescence spectroscopy

Three-dimensional fluorescence spectroscopy (excitation–emission matrix, EEM) was employed to further evaluate structural and microenvironmental changes in HSA upon interaction with TMH-IL salt. The EEM contour map of native HSA (10 µM) exhibited a dominant fluorescence peak (Peak 1) located at excitation wavelengths of approximately 275–280 nm and emission wavelengths of ∼335–340 nm (Fig. S2, SI), corresponding mainly to the intrinsic emission of Trp-214, the primary fluorophore of the protein.

When HSA was mixed with TMH-IL salt (40 µM), a noticeable decrease in the fluorescence intensity of Peak 1 was observed without significant displacement of the spectral position. This reduction in fluorescence intensity indicates perturbation of the microenvironment surrounding the Trp residue upon ligand binding. The absence of additional emission bands suggests that the interaction does not generate new fluorescent species but rather quenches the intrinsic fluorescence of HSA.

In addition to Peak 1, a diagonal band labelled a, was observed in the EEM spectra, corresponding to first-order Rayleigh scattering (*λ*_ex_ = *λ*_em_). This feature arises from optical scattering effects and does not represent genuine fluorescence emission of the protein.

All measurements were performed under low absorbance conditions (*A*_ex_ + *A*_em_ < 0.2) to minimize inner filter effects. The observed decrease in the intensity of Peak 1, together with the absence of significant spectral shifts, supports an interaction between TMH-IL salt and HSA and is consistent with the predominantly static quenching behavior inferred from the steady-state fluorescence analysis.

### 3.6 Comparison of docking and MD interaction profiles

**Fig. 10:**
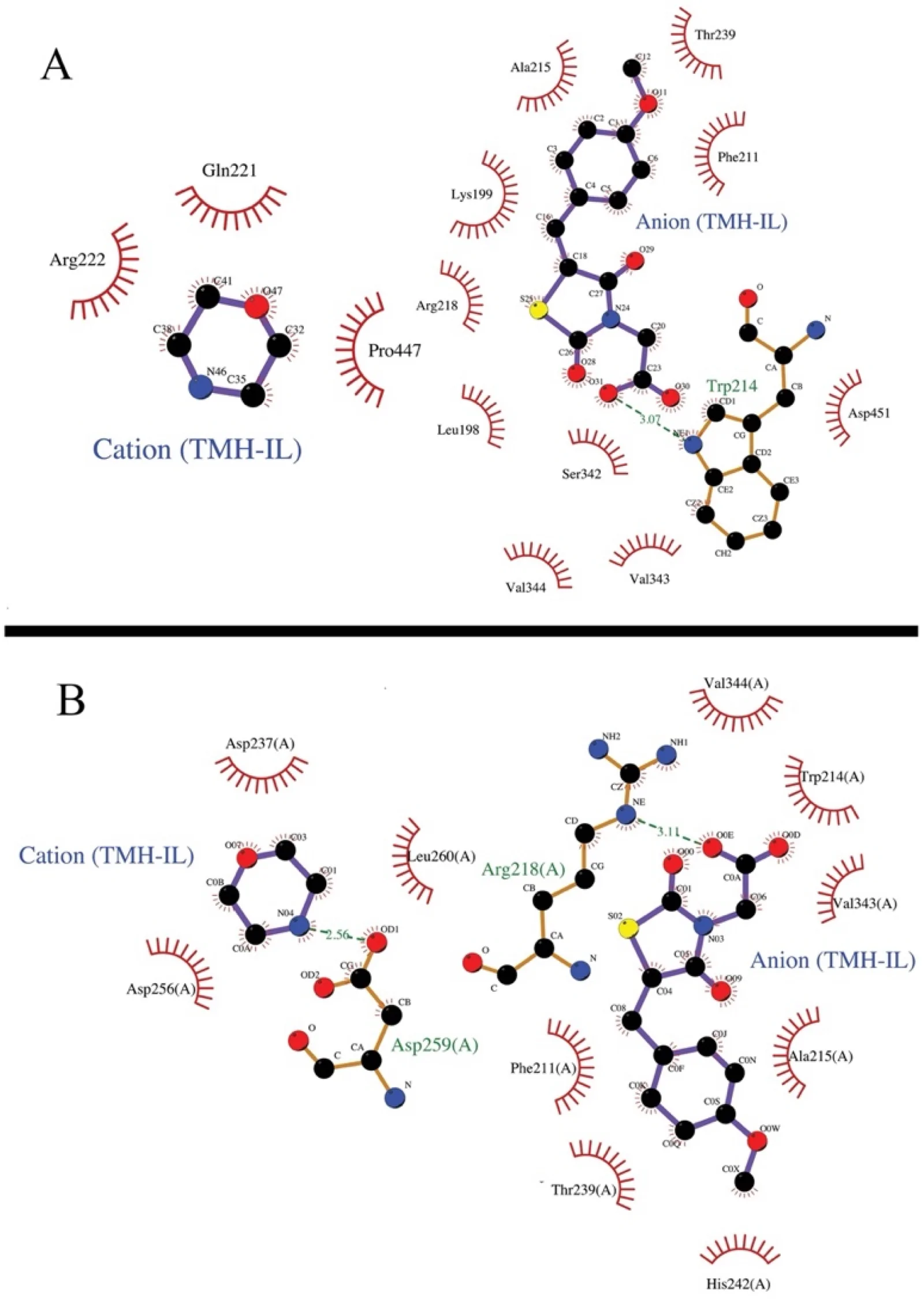
Two-dimensional LigPlot interaction maps of the HSA-TMH-IL complex obtained from (A) the top-ranked docking pose and (B) an MD-derived structure. The comparison illustrates partial conservation and rearrangement of residues surrounding TMH-IL salt following MD simulation.

Comparison of the LigPlot interaction maps obtained from (A) the top-ranked docking pose and (B) the MD-derived structure revealed partial conservation of the initial binding environment.^72^ Several residues identified in the docking model, including Phe211, Trp214, Ala215, Arg218, Thr239, Val343, and Val344, were also observed in the MD-derived interaction profile, while additional contacts were identified in the MD-derived structure. These results indicate rearrangement of the interaction network following MD simulation while retaining key features of the initially predicted binding region (Fig. 10).

### 3.7 Molecular dynamics simulation

Following proper equilibration of the systems, 150 ns production molecular dynamics (MD) simulations were performed for both free HSA and the HSA-TMH-IL complex to investigate their time-dependent structural behaviour under near-physiological conditions.^73^ The resulting trajectories were subsequently analysed comparatively to assess the conformational stability and dynamic properties of HSA in the absence and presence of TMH-IL salt.

Several structural descriptors were evaluated, including the root mean square deviation (RMSD) of backbone atoms to monitor global structural stability, the radius of gyration (*R*_g_) to assess overall compactness, and the root mean square fluctuation (RMSF) to characterize residue-level flexibility.^74^ In addition, solvent-accessible surface area (SASA), secondary-structure evolution, and hydrogen-bonding interactions were analysed.

#### 3.7.1 Secondary structure analysis

Comparison of the secondary structures of free HSA and the HSA-TMH-IL complex (Table 6) indicates that ligand binding induces limited conformational rearrangement without substantially disrupting the overall architecture of HSA. HSA remains predominantly α-helical after ligand binding.^75^ The most pronounced change is a reduction in α-helix content from 51.292% in free HSA to 48.715% in the HSA-TMH-IL complex, corresponding to a decrease of 2.577 percentage points. In contrast, the β-sheet content remains essentially unchanged (0.002% and 0.000% for free HSA and the complex, respectively), indicating that the decrease in α-helical content is not accompanied by a major α-helix-to-β-sheet transition.

**Table 6:** Secondary structure content (%) of free HSA and the HSA-TMH-IL complex obtained from molecular dynamics simulations

Secondary structure	HSA-TMH-IL (%)	Free HSA (%)	*Δ* = Complex − free
α-Helix	48.715	51.292	−2.577
β-Sheet	0.000	0.002	−0.002
Turn	16.089	15.908	+0.181
Bend	13.670	12.488	+1.182
3_10_-helix	4.888	4.048	+0.840
π-Helix	0.220	0.300	−0.080
PPII	2.104	2.259	−0.155
Coil	14.311	13.703	+0.608

The reduction in α-helical content is accompanied by increases in Bend from 12.488% to 13.670% (*Δ* = +1.182%), 3_10_-helix from 4.048% to 4.888% (*Δ* = +0.840%), Coil from 13.703% to 14.311% (*Δ* = +0.608%), and Turn from 15.908% to 16.089% (*Δ* = +0.181%). These relatively modest changes indicate redistribution of secondary-structure elements and local backbone rearrangement upon TMH-IL salt binding rather than extensive structural disruption.^76,77^

The MD results are consistent with the experimental spectroscopic observations. FTIR deconvolution showed a modest decrease in α-helical content from 62.03% to 60.16%, while the CD spectra showed only small reductions in ellipticity at 208 and 222 nm without appreciable shifts in the characteristic α-helical bands. Furthermore, the change in the microenvironment of Trp214 observed by 3D fluorescence supports a localized structural response to TMH-IL salt binding. Although the absolute secondary-structure values obtained from computational and spectroscopic approaches are not directly comparable, the consistent trends indicate that TMH-IL salt induces limited local conformational adaptation while largely preserving the predominantly α-helical architecture and overall structural integrity of HSA.

The values were calculated as the average percentages of secondary-structure elements (α-helix, β-sheet, 3_10_-helix, π-helix, PPII, bend, turn, and coil) over the final 130 ns (20–150 ns) of the MD trajectories using the DSSP algorithm.

#### 3.7.2 Structural stability and dynamics of the HSA-TMH-IL complex

**Fig. 11:**
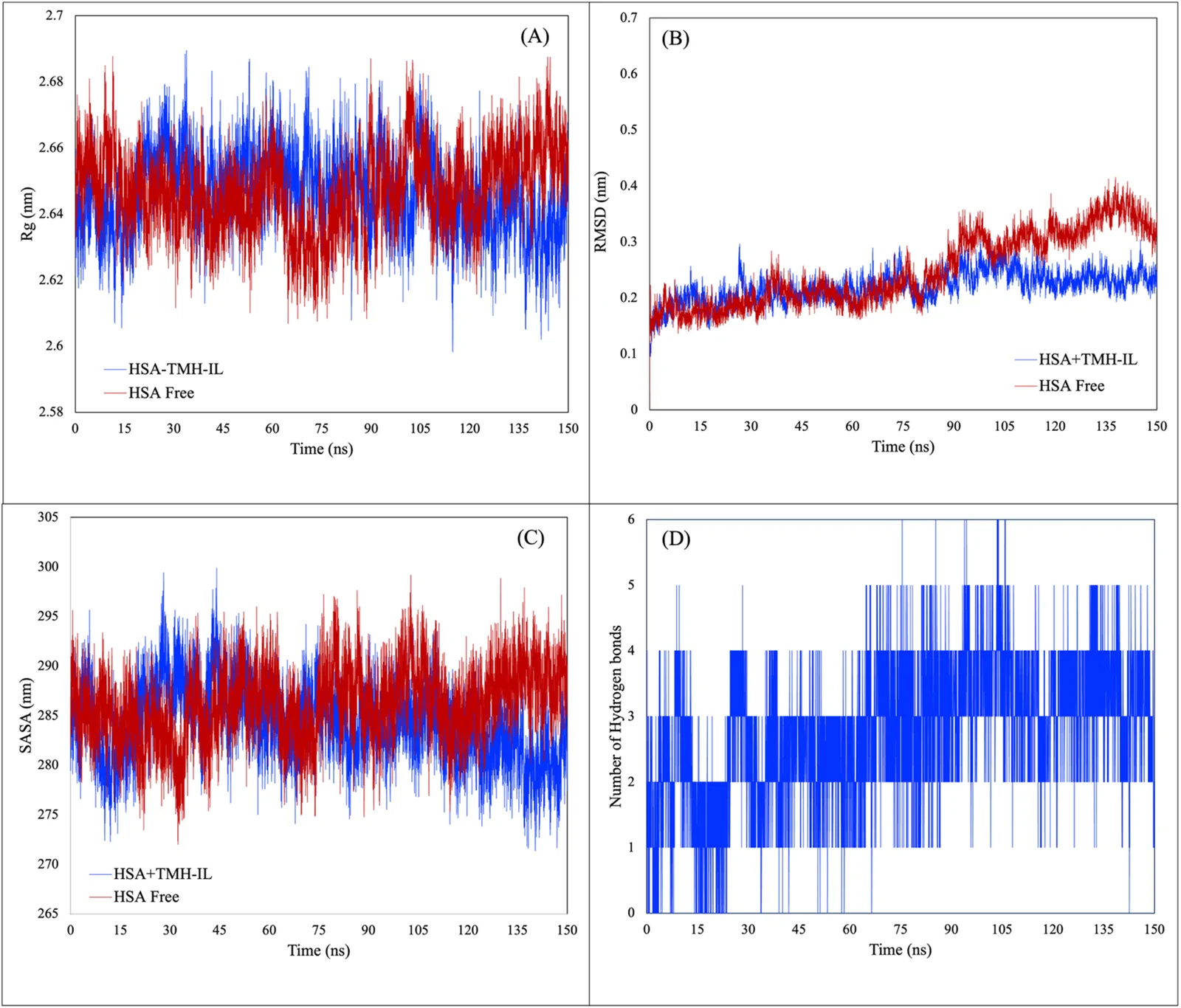
Structural stability analysis of the HSA-TMH-IL complex compared with free HSA during the 150 ns MD simulations. (A) Radius of gyration (*R*_g_), (B) root mean square deviation (RMSD) of the protein backbone atoms, (C) solvent-accessible surface area (SASA), and (D) number of hydrogen bonds formed between HSA and TMH-IL salt during the simulation. The reported profiles represent the mean values obtained from three independent MD simulations.

As shown in Fig. 11, comparison of the dynamic behavior of free HSA and the HSA-TMH-IL complex based on RMSD, *R*_g_, and SASA indicates that TMH-IL salt binding does not substantially perturb the overall structure of HSA.^78^ At the beginning of the simulation, the RMSD of both systems gradually increases, reflecting relaxation from their initial structures and adaptation to the simulation conditions.^79^ Subsequently, the RMSD of the HSA-TMH-IL complex remains within a relatively stable range, whereas free HSA exhibits somewhat greater fluctuations toward the end of the trajectory. These observations indicate that TMH-IL salt binding does not induce pronounced structural destabilization of HSA.

The *R*_g_ values of both systems remain approximately within the range of 2.61–2.66 nm, without a clear increasing or decreasing trend, indicating preservation of the overall compactness of HSA upon ligand binding. Similarly, the SASA values of both systems fluctuated predominantly predominantly within the range of 280–295 nm^2^. During the latter part of the trajectory, the HSA-TMH-IL complex tended to exhibit somewhat lower SASA values than free HSA, which may reflect localized conformational rearrangements accompanied by reduced solvent exposure. Overall, the RMSD, *R*_g_, and SASA profiles indicate that TMH-IL salt binding is associated with limited conformational adaptation while largely preserving the overall architecture and compactness of HSA.^78,80^

Hydrogen-bond analysis further revealed that the HSA-TMH-IL interaction network remained dynamic throughout the 150 ns trajectory. The number of direct protein–ligand hydrogen bonds varied over time, indicating continuous rearrangement of individual contacts. Notably, residue-based occupancy analysis identified Arg218 as a particularly persistent hydrogen-bonding residue, with an occupancy of approximately 91.74% over the analyzed trajectory. Additional hydrogen-bonding contacts occurred with different occupancies, indicating that the interaction network was continuously reorganized rather than remaining fixed in a single geometry. The persistence of the Arg218 interaction is particularly noteworthy because this residue was also involved in hydrogen-bond formation in the initial docking model, thereby providing a direct connection between the docking prediction and the dynamic MD behavior.

Taken together, the RMSD, *R*_g_, SASA, and hydrogen-bond analyses indicate that TMH-IL salt binding is accompanied by dynamic and localized rearrangements without major disruption of the overall HSA structure. These observations are consistent with the spectroscopic results, which indicated preservation of the global protein architecture despite limited ligand-induced conformational changes.

The RMSF profiles of free HSA and the HSA-TMH-IL complex showed broadly similar residue-wise fluctuation patterns (Fig. S3 in the SI). However, the complex generally exhibited lower or comparable RMSF values, with attenuation of several fluctuation peaks observed in free HSA. This behavior suggests that TMH-IL salt binding locally restricts residue mobility without substantially altering the overall flexibility pattern of HSA, consistent with reduced local conformational fluctuations upon ligand binding.

### 3.8 Molecular docking analysis

Molecular docking simulations were performed using the GOLD 2022.3 software package to investigate the binding behavior of TMH-IL salt within the principal drug-binding regions of HSA. Considering the ionic nature of TMH-IL salt and its expected dissociation in aqueous media, three docking systems were examined: the anionic species, the cationic species, and the co-docked ion pair (anion + cation). This strategy was employed to assess the individual binding contribution of each ionic component as well as their possible cooperative behavior within the HSA binding pockets.

Docking calculations were carried out at the three major ligand-binding regions of HSA: Sudlow's site I (subdomain IIA), Sudlow's site II (subdomain IIIA), and site III (subdomain IB). For each binding form and binding site, the generated poses were ranked using the ChemPLP scoring function. The PLP ligand energy was also considered as an additional energetic descriptor for comparison of the docked conformations. The highest-scoring pose obtained for each docking system was selected for subsequent structural analysis. Comparison of the docking results for the three binding forms and the three investigated HSA sites is presented in Table 7. The ChemPLP scores and PLP ligand energies obtained for the three binding forms at the investigated HSA binding sites are summarized in Table 7.

**Table 7:** Comparative docking scores of the anionic, cationic, and co-docked ligand forms within Sudlow's sites I (subdomain IIA), II (subdomain IIIA), and III (subdomain IB) of HSA. The best-ranked poses were selected according to obtain the highest PLP fitness score

Site	Binding form	ChemPLP (PLP fitness)	PLP ligand energy
Site I	Anion	63.77	−56.00
	Cation	26.48	−20.48
	Co-docked (anion + cation)	71.07	−69.44
Site II	Anion	48.83	−34.92
	Cation	20.80	−17.80
	Co-docked (anion + cation)	48.42	−43.78
Site III	Anion	55.14	−42.58
	Cation	23.78	−16.10
	Co-docked (anion + cation)	64.61	−54.06

Unlike the parent 2,4-thiazolidinedione, which preferentially binds within subdomain IB, TMH was predicted to occupy Sudlow's site I, similarly to pioglitazone. This difference indicates that structural modification of the thiazolidinedione scaffold influences the preferred binding pocket on HSA.

#### 3.8.1 Binding preference across HSA sites

Among the investigated binding pockets, Sudlow's site I (subdomain IIA) exhibited the most favorable docking results for TMH-IL salt (Table 7). Comparison of the three docking systems showed that the co-docked anion–cation system yielded the highest ChemPLP score (71.07), followed by the anionic species (63.77), whereas the cationic species alone showed a considerably lower score (26.48). The corresponding PLP ligand energy for the co-docked system was −69.44, further supporting favorable accommodation of the two ionic components within this binding cavity.

At site III (subdomain IB), the co-docked system also exhibited a relatively high ChemPLP score of 64.61, suggesting that this region may represent a secondary favorable binding site. In contrast, site II (subdomain IIIA) showed comparatively lower docking scores, with the co-docked system and the anionic species giving ChemPLP scores of 48.42 and 48.83, respectively.

Overall, based on the ChemPLP scores obtained for the co-docked systems, the preference of the investigated HSA binding regions followed the order:

Site I > Site III > Site II.

The higher ChemPLP score obtained for the co-docked anion–cation system at site I compared with either ionic species docked individually suggests that simultaneous accommodation of both ionic components may provide favorable cooperative interactions within the binding pocket. Nevertheless, considering the expected dissociation of TMH-IL salt in aqueous solution, the co-docking results should be interpreted as a computational assessment of the possible simultaneous presence of the two ionic components within the HSA binding cavity rather than evidence for the persistence of an intact ion pair in solution.

#### 3.8.2 Interaction analysis in Sudlow's site I

**Fig. 12:**
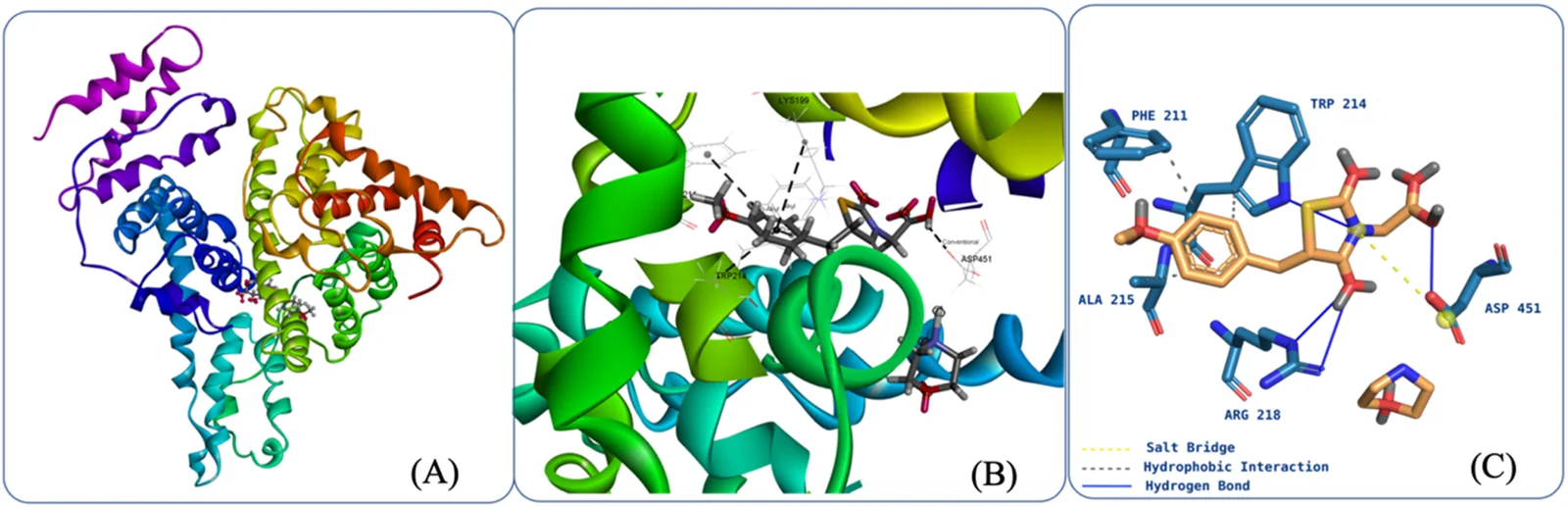
Predicted binding conformation of TMH-IL salt within HSA obtained from GOLD docking: (A) overall structure of the HSA–TMH-IL complex, (B) enlarged view of the binding pocket visualized using Discovery Studio, and (C) three-dimensional protein–ligand interaction profile generated using PLIP.

Detailed interaction analysis of the top-ranked co-docked pose at Sudlow's site I revealed several non-covalent interactions between the ionic components of TMH-IL salt and surrounding HSA residues (Fig. 12 and Table S1 in SI). PLIP analysis identified hydrophobic contacts with Phe211, Trp214, and Ala215 at distances of 3.52, 3.69, and 3.52 Å, respectively. Hydrogen-bonding interactions were also observed with Trp214, Arg218, and Asp451. Notably, two hydrogen-bond contacts were detected with Arg218, whereas Asp451 participated in both hydrogen bonding and a salt-bridge interaction.

The involvement of Trp214, the principal intrinsic fluorophore of HSA, is consistent with the experimentally observed perturbation and quenching of HSA fluorescence upon addition of TMH-IL salt. Overall, the docking and PLIP results suggest that hydrophobic, hydrogen-bonding, and electrostatic interactions may collectively contribute to the favorable accommodation of the ionic components within Sudlow's site I.

#### 3.8.3 Interpretation of docking results

The occurrence of favorable docking poses across multiple HSA binding regions reflects the ability of HSA to accommodate TMH-IL salt within different binding pockets. Nevertheless, the higher ChemPLP score obtained for the co-docked system at Sudlow's site I indicates that this cavity represents the most favorable binding region among the sites investigated in the present docking calculations.

Interestingly, this binding-site preference differs from that previously reported for the parent 2,4-thiazolidinedione, which was predicted to bind preferentially within subdomain IB of HSA. In contrast, the present docking results identify Sudlow's site I (subdomain IIA) as the most favorable binding region for TMH-IL salt. This difference suggests that structural modification of the thiazolidinedione scaffold, together with the presence of the morpholinium ionic component, may alter the molecular recognition pattern toward HSA and consequently influence the preferred binding region.

Overall, the docking results support favorable accommodation of the ionic components of TMH-IL salt within Sudlow's site I. The higher ChemPLP score obtained for the co-docked system compared with the individually docked ionic species suggests that simultaneous accommodation of the anionic and cationic components may provide additional favorable interactions within the binding pocket. These results should, however, be interpreted as a computational assessment of possible cooperative interactions within the HSA binding site rather than evidence for the persistence of an intact ion pair in aqueous solution.

## 4 Conclusion

In this study, the interaction between the thiazolidinedione-morpholine-based ionic liquid salt (TMH-IL) and human serum albumin (HSA) was investigated using a combination of spectroscopic and computational approaches. Fluorescence measurements indicated significant quenching of the intrinsic emission of HSA, and temperature-dependent Stern–Volmer analysis suggested that the quenching process predominantly follows a static mechanism. Thermodynamic parameters revealed a spontaneous and enthalpy-driven binding process. FT-IR and CD analyses demonstrated only minor perturbations in the secondary structure of HSA, indicating that TMH-IL salt binding does not induce extensive unfolding or denaturation of the protein.

Molecular docking simulations predicted Sudlow's site I (subdomain IIA) as the preferred binding region of TMH-IL salt. Structural analysis indicated that the interaction is stabilized by a combination of hydrophobic contacts and hydrogen-bonding interactions involving key residues within the binding cavity. Comparative molecular dynamics simulations of free HSA and the HSA-TMH-IL complex further showed that ligand binding largely preserves the overall structural stability and compactness of HSA, while persistent interactions with key binding-site residues supported the persistence of protein–ligand contacts throughout the simulations.

Overall, the integrated spectroscopic, docking, and MD results indicate that TMH-IL salt associates with HSA while largely preserving the native structural organization of the protein. The preferential recognition of Sudlow's site I and the limited ligand-induced conformational changes provide molecular-level insight into the potential transport and distribution behavior of TMH-IL salt.

## Author contributions

Golshan Golshanian: conceptualization, methodology, investigation, data curation, formal analysis, visualization, writing – original draft. Mohammad Mehdi Alavianmehr: methodology, validation, investigation (molecular docking and molecular dynamics), review & editing, funding acquisition. Mohammad Navid Soltani Rad: supervision, conceptualization, writing – review and editing, funding acquisition. Somayeh Behrouz: synthesis, purification and characterization. Mohsen Sorouri: solution preparation & analysis and Delara Mohammad Aghaie: methodology, validation, investigation (physical chemistry aspects including docking and dynamics).

## Conflicts of interest

The authors declare that they have no known competing financial interests or personal relationships that could have appeared to influence the work reported in this paper.

## Note added after first publication

This article replaces the version published on 27 August 2026 which included errors in the captions for Fig. 11 and 12.

## Supplementary Material

RA-016-D6RA05468D-s001

## Data Availability

As the corresponding author, and on behalf of my co-authors, i would like to state that, the data supporting this paper is available upon request. Supplementary information (SI): the fluorescence emission spectrum of TMH-IL salt, the three-dimensional fluorescence excitation-emission matrix (EEM) contour map of HSA in the presence of TMH-IL salt, residue-wise RMSF profiles of free HSA and the HSA-TMH-IL complex, and PLIP-derived protein–ligand interaction data for the top-ranked docking pose. See DOI: https://doi.org/10.1039/d6ra05468d.
