# Supplementary material for: Molecular recognition of a Novel thiazolidinedione–morpholine-based ionic liquid salt by HSA: spectroscopic and molecular dynamics analysis
Source: RSC Adv. 2026 Aug 27;16(42):50911–30. doi: 10.1039/d6ra05468d (PMC13521121; doi:10.1039/d6ra05468d)
Supplement: Supplementary file 1 [file RA-016-D6RA05468D-s001.pdf]

Supplementary Information (SI) for RSC Advances.
This journal is © The Royal Society of Chemistry 2026

RSC Advances

(Electronic Supplementary Information)

Molecular Recognition of a Novel Thiazolidinedione–Morpholine-Based Ionic Liquid Salt by HSA: Spectroscopic and Molecular Dynamics Analysis

Golshan Golshanian, Mohammad Mehdi Alavianmehr*, Mohammad Navid Soltani Rad*, Somayeh Behrouz, Mohsen Sorouri, Delara Mohammad-Aghaie,

Department of Chemistry, Shiraz University of Technology, Shiraz 71555-313, Iran

Tel.: +98 71 3735 4500; Fax: +98 71 3735 4520

E-mail address: alavianmehr@sutech.ac.ir (M.M. Alavianmehr), soltani@sutech.ac.ir (M.N.Soltani Rad).

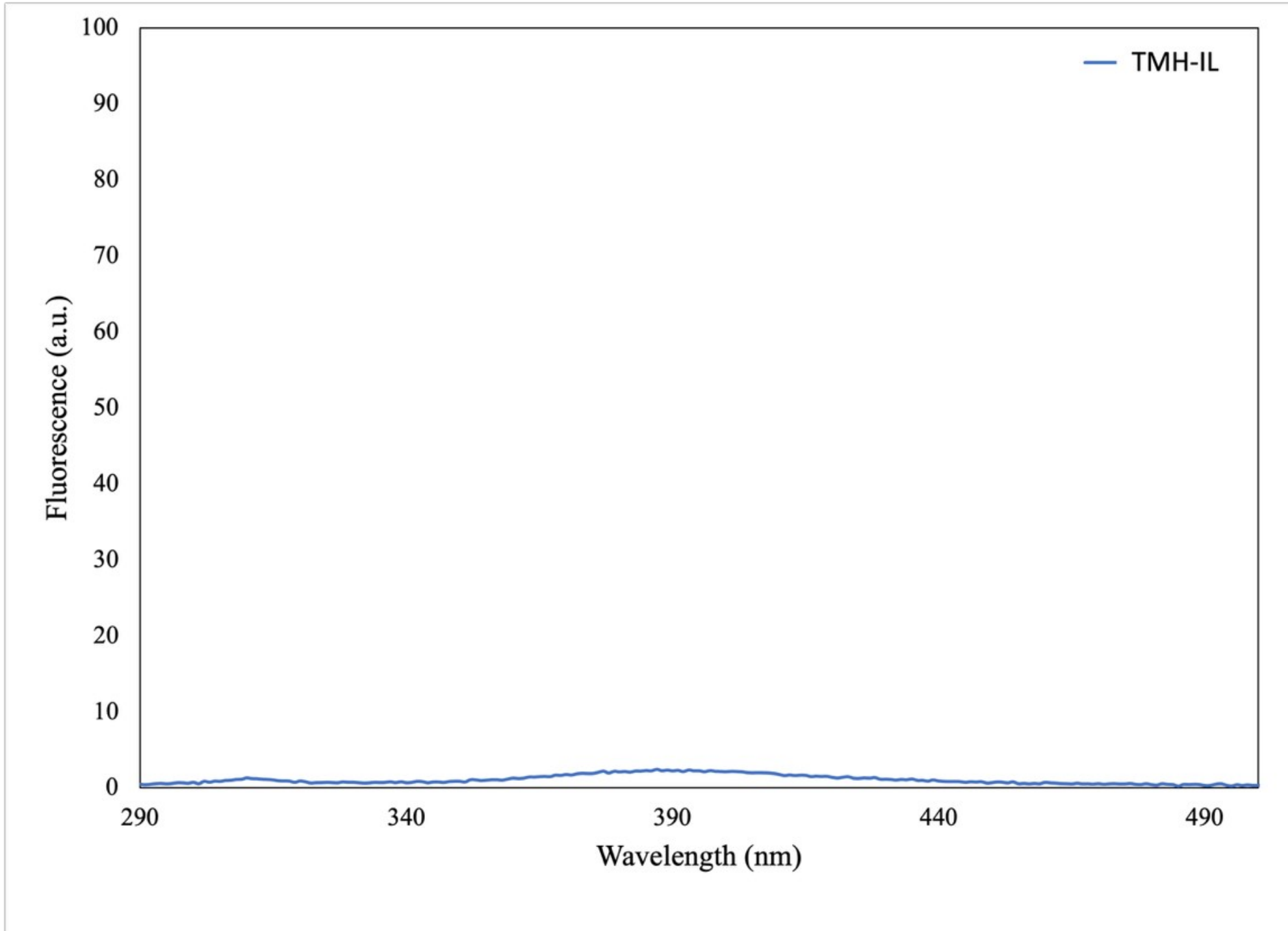

Fig. S1 Fluorescence emission spectrum of TMH-IL salt alone under identical experimental conditions.

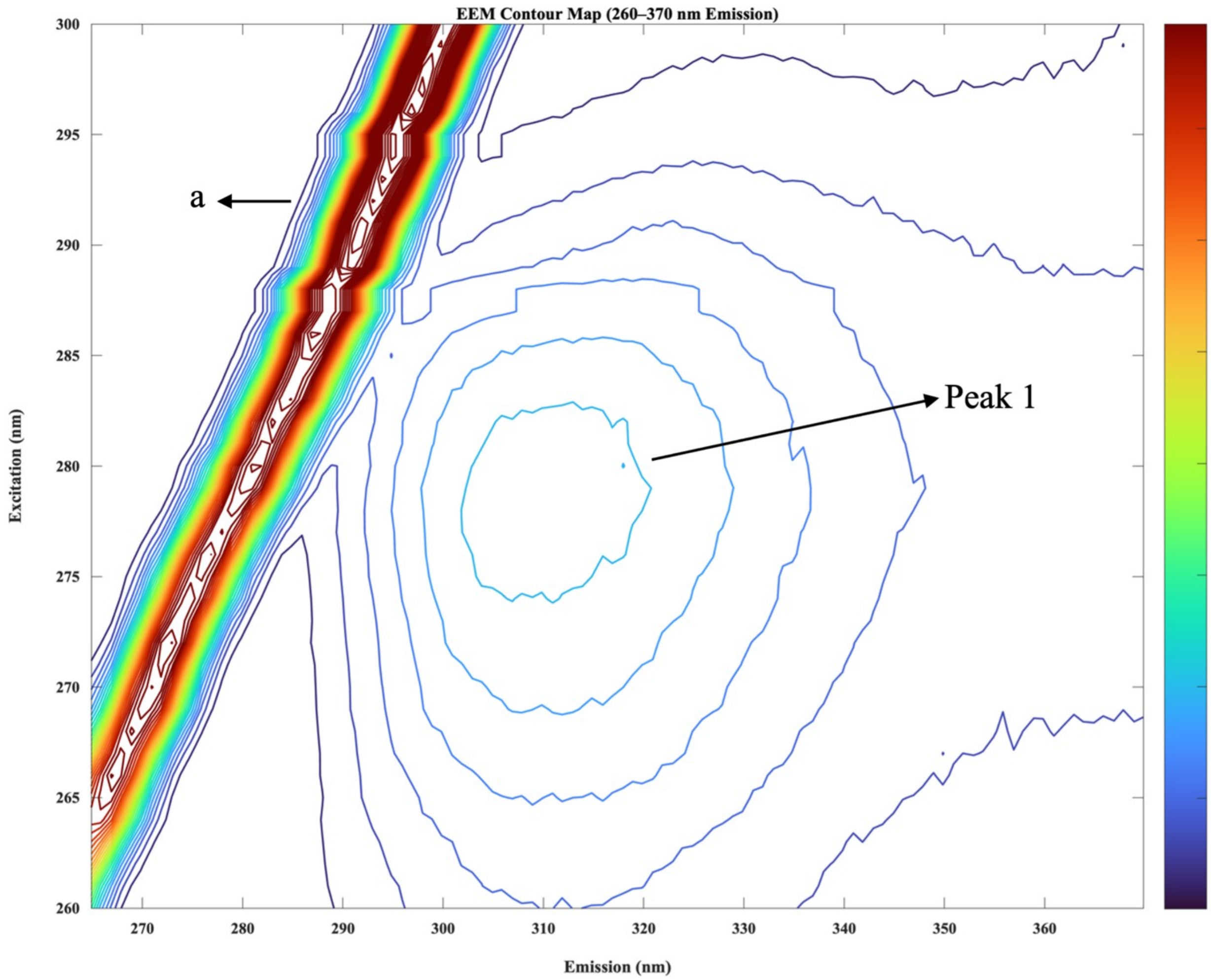

Fig. S2 Three-dimensional fluorescence excitation–emission matrix (EEM) contour map of HSA (10 μM) in the presence of TMH-IL salt (40 μM), showing quenching of the intrinsic Trp-214 fluorescence.

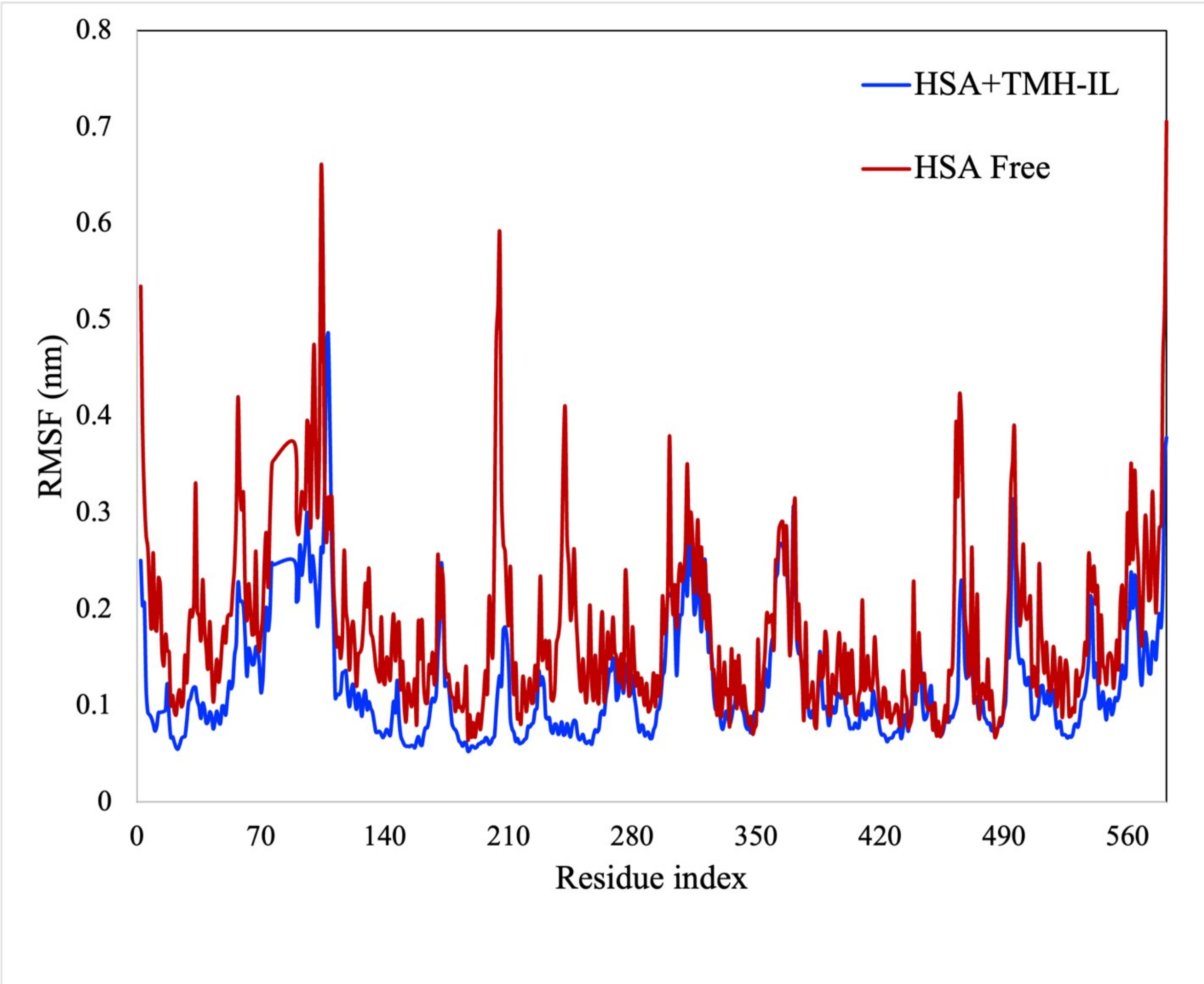

Fig. S3 Residue-wise root mean square fluctuation (RMSF) profiles of free HSA and the HSA–TMH-IL complex during the 150 ns MD simulations.

Table S1. Protein–ligand interactions between TMH-IL salt and HSA identified by PLIP analysis for the top-ranked docking pose.

Interaction type	HSA residue	Distance (Å)
Hydrophobic	Phe211	3.52
Hydrophobic	Trp214	3.69
Hydrophobic	Ala215	3.52
Hydrogen bond	Trp214	2.84
Hydrogen bond	Arg218	2.63
Hydrogen bond	Arg218	3.24
Hydrogen bond	Asp451	2.20
Salt bridge	Asp451	5.16
